# The small RNA mascRNA differentially regulates TLR-induced proinflammatory and antiviral responses

**DOI:** 10.1172/jci.insight.150833

**Published:** 2021-11-08

**Authors:** Tao Sun, Chunxue Wei, Daoyong Wang, Xuxu Wang, Jiao Wang, Yuqing Hu, Xiaohua Mao

**Affiliations:** 1 School of Life Science and Technology, Key Laboratory of Ministry of Education for Developmental Genes and Human Disease,; 2 Department of Biochemistry and Molecular Biology, School of Medicine,; and 3 Jiangsu Provincial Key Laboratory of Critical Care Medicine, Southeast University, Nanjing, Jiangsu, China.

**Keywords:** Immunology, Cytokines, Innate immunity, Noncoding RNAs

## Abstract

MALAT1-associated small cytoplasmic RNA (mascRNA) is a highly conserved transfer RNA–like (tRNA-like) noncoding RNA whose function remains largely unknown. We show here that this small RNA molecule played a role in the stringent control of TLR-mediated innate immune responses. mascRNA inhibited activation of NF-**κ**B and mitogen-activated protein kinase (MAPK) signaling and the production of inflammatory cytokines in macrophages stimulated with LPS, a TLR4 ligand. Furthermore, exogenous mascRNA alleviated LPS-induced lung inflammation. However, mascRNA potentiated the phosphorylation of IRF3 and STAT1 and the transcription of IFN-related genes in response to the TLR3 ligand poly(I:C) both in vitro and in vivo. Mechanistically, mascRNA was found to enhance K48-linked ubiquitination and proteasomal degradation of TRAF6, thereby negatively regulating TLR-mediated MyD88-dependent proinflammatory signaling while positively regulating TRIF-dependent IFN signaling. Additionally, heterogeneous nuclear ribonucleoprotein H (hnRNP H) and hnRNP F were found to interact with mascRNA, promote its degradation, and contribute to the fine-tuning of TLR-triggered immune responses. Taken together, our data identify a dual role of mascRNA in both negative and positive regulation of innate immune responses.

## Graphical abstract

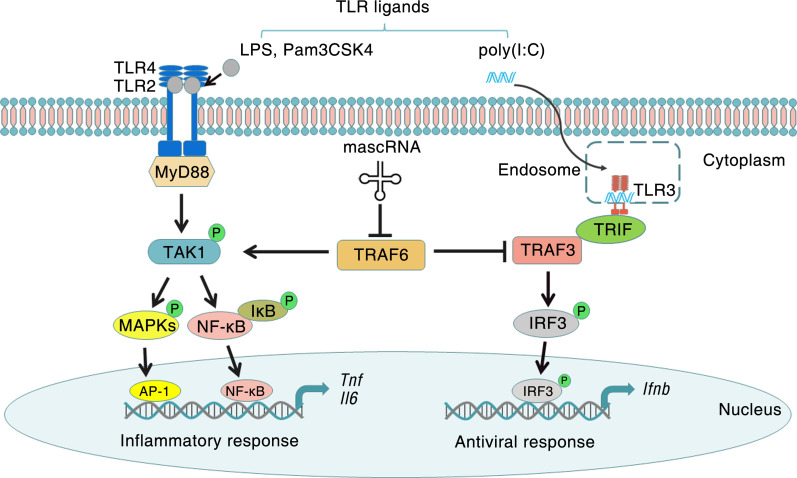

## Introduction

TLRs are evolutionarily ancient pattern-recognition receptors that function in innate immunity against pathogens. TLR signaling is generally classified by their dependence on 2 adaptor proteins: MyD88, which is essential for signaling from all TLRs except TLR3, and TRIF, which is essential for TLR3/4 signaling (1, 2). In the MyD88-dependent pathway, a supramolecular organizing center (SMOC) called myddosome is assembled upon recognizing a pathogen-associated molecular pattern by a TLR. Myddosome, with MyD88 and TRAF6 as its core components, activates TGF-β–activated kinase 1–dependent (TAK1-dependent) NF-κB and mitogen-activated protein kinase (MAPK) signaling, leading to the induction of proinflammatory cytokines and glycolysis (3). In the TRIF-dependent pathway, the putative triffosome, with TRIF and TRAF6/3 as its core components, functions as a SMOC and activates the kinase TBK1, leading to the expression of type I IFNs and also necroptosis upon caspase inhibition (3).

TRAF6 is a central adaptor in TLR signaling pathways. In myddosome, MyD88 interacts with TRAF6 via members of the IRAK family, and this promotes TRAF6 homo-oligomization and induces its E3 ubiquitin ligase activity. TRAF6 then functions with an E2 ubiquitinligase complex of Ubc13 and Uev1A to generate K63-linked polyubiquitin chains on itself and other proteins (1, 3–5). These ubiquitin chains act as a scaffold to recruit and activate downstream kinase complexes containing, for instance, TAK1. In the triffosome, TRIF recruits TRAF6 and TRAF3, leading to TRAF6-dependent activation of NF-κB and TRAF3-dependent transcriptional activation of type I IFNs (1, 3). Thus, as a key adaptor protein in inflammatory signaling pathways, TRAF6 activity must be tightly regulated. Several deubiquitinases, such as CYLD, A20, Otud7b, USP2a, USP4, USP20, and MYSM1, are known to inhibit TRAF6 activity by directly removing its K63-linked ubiquitin chains (6). Two ubiquitin ligases TRIM38 and RNF19A, on the other hand, catalyze K48-linked ubiquitination of TRAF6 for proteasomal degradation (7, 8). Furthermore, 2 kinases, MST4 and DRAK1, prevent TRAF6 autoubiquitination by blocking its oligomerization in a kinase activity-dependent or -independent manner (9, 10). In contrast, RSK2, a kinase of the RSK family, phosphorylates TRAF6 and, as a result, induces its K63 ubiquitination (11). Regulation of TRAF6 also involves other proteins. UBE2O, a putative E2 ubiquitin-conjugating enzyme, binds to TRAF6 to inhibit its K63-polyubiquitination (12); HOPS, a transmembrane ubiquitin-like protein, binds to TRAF6 to inhibit its proteasomal degradation (13); and NLRC3, a member of the NLR family of sensors, associates with TRAF6 and diminishes its K63-linked ubiquitination (14). Besides protein factors, TRAF6 is subject to regulation through noncoding RNA. For instance, a long noncoding RNA (lncRNA) named Mirt2 binds TRAF6 and inhibits its oligomerization and autoubiquitination (15), while 2 microRNAs, miR-125 and miR-124, target *TRAF6* 3′UTR for degradation (16).

MALAT1-associated small cytoplasmic RNA (mascRNA) is a highly conserved transfer RNA–like (tRNA-like) small noncoding RNA with a size of 61 nucleotides (17). It is derived from posttranscriptional processing of the precursor of a lncRNA named MALAT1. Processing at the 3′-end of pre-MALAT1 transcript by RNases P and Z simultaneously generates mature MALAT1 and mascRNA, which are nucleus and cytoplasm localized, respectively (17). MALAT1 has been demonstrated to participate in cancer progression (18), as well as in other cellular processes like the modulation of LPS-induced cytokine expression (19, 20). At present, the function of mascRNA remains largely unknown. The only role so far assigned to mascRNA is its association with immunoregulation, based on the observations that ablation of mascRNA in THP-1 monocytes influences immune gene expression and exogenous mascRNA renders cardiomyocytes resistant to CVB3 infection (21). However, whether and how mascRNA influences TLR signaling is unclear. Here, we provide evidence that mascRNA, whose abundance is influenced by heterogeneous nuclear ribonucleoprotein H (hnRNP H) and hnRNP F (hnRNP H/F), promotes K48-linked ubiquitination of TRAF6 to fine-tune TLR-induced innate immune responses.

## Results

### mascRNA suppresses LPS-induced proinflammatory cytokine production.

**Figure 1:**
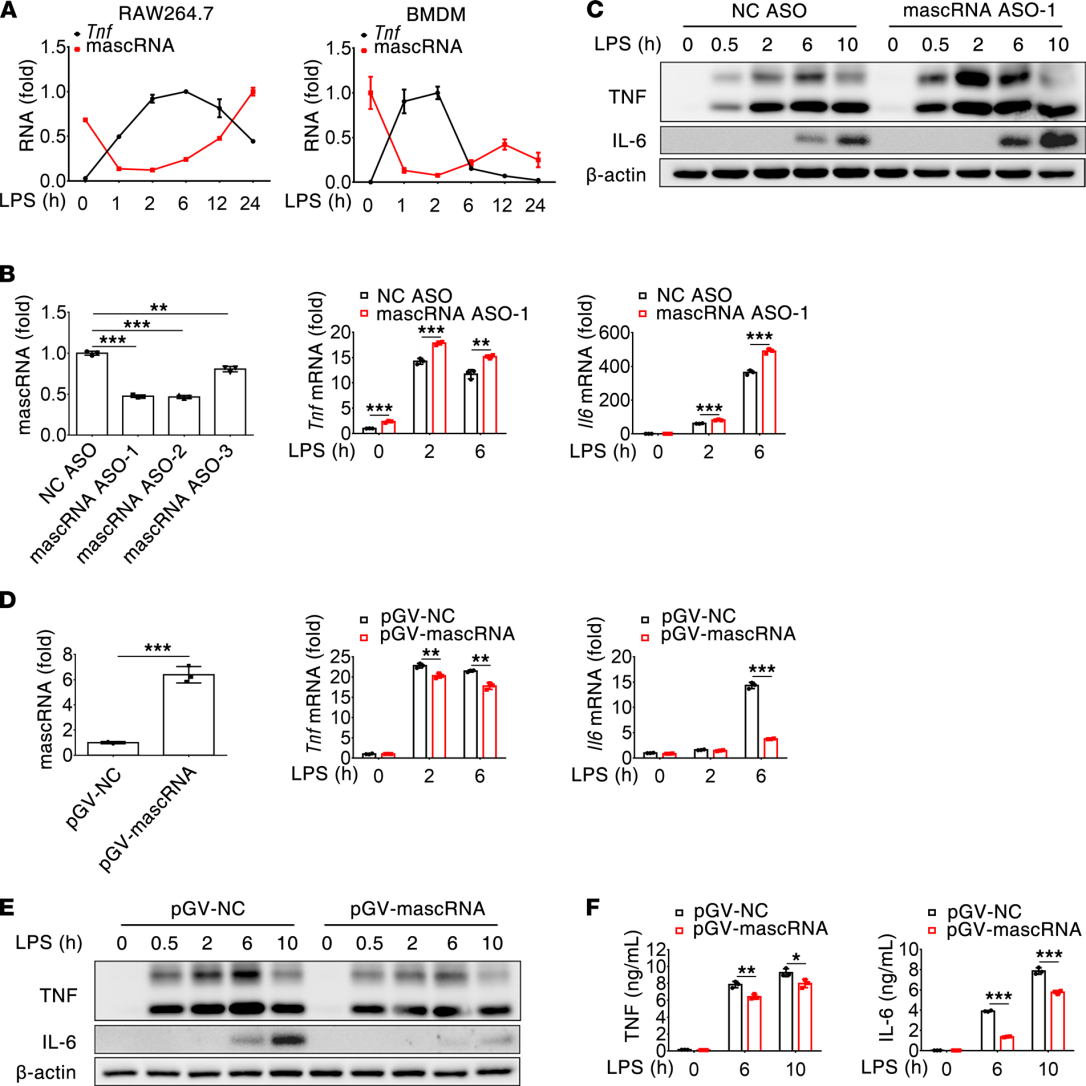
mascRNA inhibits proinflammatory cytokine production. (**A**) Kinetics of mascRNA and *Tnf* expression in LPS-stimulated mouse RAW264.7 cells and BMDMs. RNA was quantified by qPCR. (**B**) mascRNA knockdown increases *Tnf* and *Il6* mRNA expression in response to LPS challenge. RAW264.7 cells were transfected with a specific antisense oligonucleotide targeting mouse mascRNA (mascRNA ASO) for 36 hours. Then, cells were stimulated with LPS for the indicated times. The knockdown efficiency of 3 mascRNA ASOs (left panel) and the mRNA abundance of *Tnf* and *Il6* were measured by qPCR. (**C**) mascRNA knockdown increases TNF and IL-6 protein expression in response to LPS challenge. RAW264.7 cells were transfected as in **B** and stimulated with LPS for the indicated times. Cellular TNF and IL-6 precursors were analyzed by immunoblot. The 2 bands detected with anti-TNF represent full-length TNF (lower band) and dimeric intermediate of TNF (upper band), respectively. (**D**–**F**) mascRNA overexpression decreases TNF and IL-6 expression in response to LPS challenge. RAW264.7 cells stably transfected with a vector overexpressing mascRNA (pGV-mascRNA) were stimulated with LPS for the indicated times. The abundance of mascRNA and *Tnf* and *Il6* mRNA were measured by qPCR (**D**), cellular TNF and IL-6 precursors analyzed by immunoblot (**E**), and secreted TNF and IL-6 measured by ELISA (**F**). NC, negative control. Data shown in **A**, **B**, **D**, and **F** are the mean ± SD of triplicate wells. *P < 0.05; **P < 0.01; ***P < 0.001 (2-tailed Student’s *t* test). Data are representatives of 2 (**F**) or 3 (**A**–**E**) independent experiments.

To investigate whether mascRNA regulates innate immune and inflammatory responses, we first determined the expression patterns of *Tnf* and mascRNA in the LPS-stimulated mouse macrophage cell line RAW264.7. We observed that *Tnf* expression was upregulated when mascRNA started to decrease within 2 hours of LPS treatment, whereas *Tnf* expression was downregulated when mascRNA started to increase 6 hours after LPS treatment (Figure 1A). Similar results were obtained in mouse BM-derived macrophages (BMDMs) (Figure 1A). The negative correlation between *Tnf* and mascRNA expression implies that mascRNA may be a protective feedback regulator limiting TLR4 signaling–induced inflammatory responses at later time points after LPS stimulation. To test this hypothesis, RAW264.7 cells were transfected with 3 antisense oligonucleotides (ASOs) targeting mascRNA, with the most efficient ones (ASO-1 and ASO-2) being selected for subsequent experiments (Figure 1B). It is worth noting that, in agreement with a previous report (21), anti-mascRNA treatment did not alter Malat1 abundance (Supplemental Figure 1A; supplemental material available online with this article; https://doi.org/10.1172/jci.insight.150833DS1). Compared with RAW264.7 cells transfected with nonspecific negative control ASO (NC ASO), knockdown of mascRNA markedly increased the expression of *Tnf* and *Il6* at both mRNA and protein levels following LPS stimulation (Figure 1, B and C). Consistently, forced expression of mascRNA resulted in reduced mRNA and intracellular protein levels of *Tnf* and *Il6*, as well as reduced secretion of TNF and IL-6 in RAW264.7 cells treated with LPS (Figure 1, D–F). The expression of TNF and IL-6 mRNA and/or protein was also increased in mascRNA-depleted human THP-1 macrophages and mouse peritoneal macrophages (MPMs) in response to LPS challenge (Supplemental Figure 1, B–E). Together, these results indicated that mascRNA negatively regulates LPS-triggered proinflammatory cytokine production in macrophages.

### mascRNA suppresses proinflammatory cytokine transcription through inhibiting MyD88/TAK1-dependent NF-κB and MAPK signaling.

**Figure 2:**
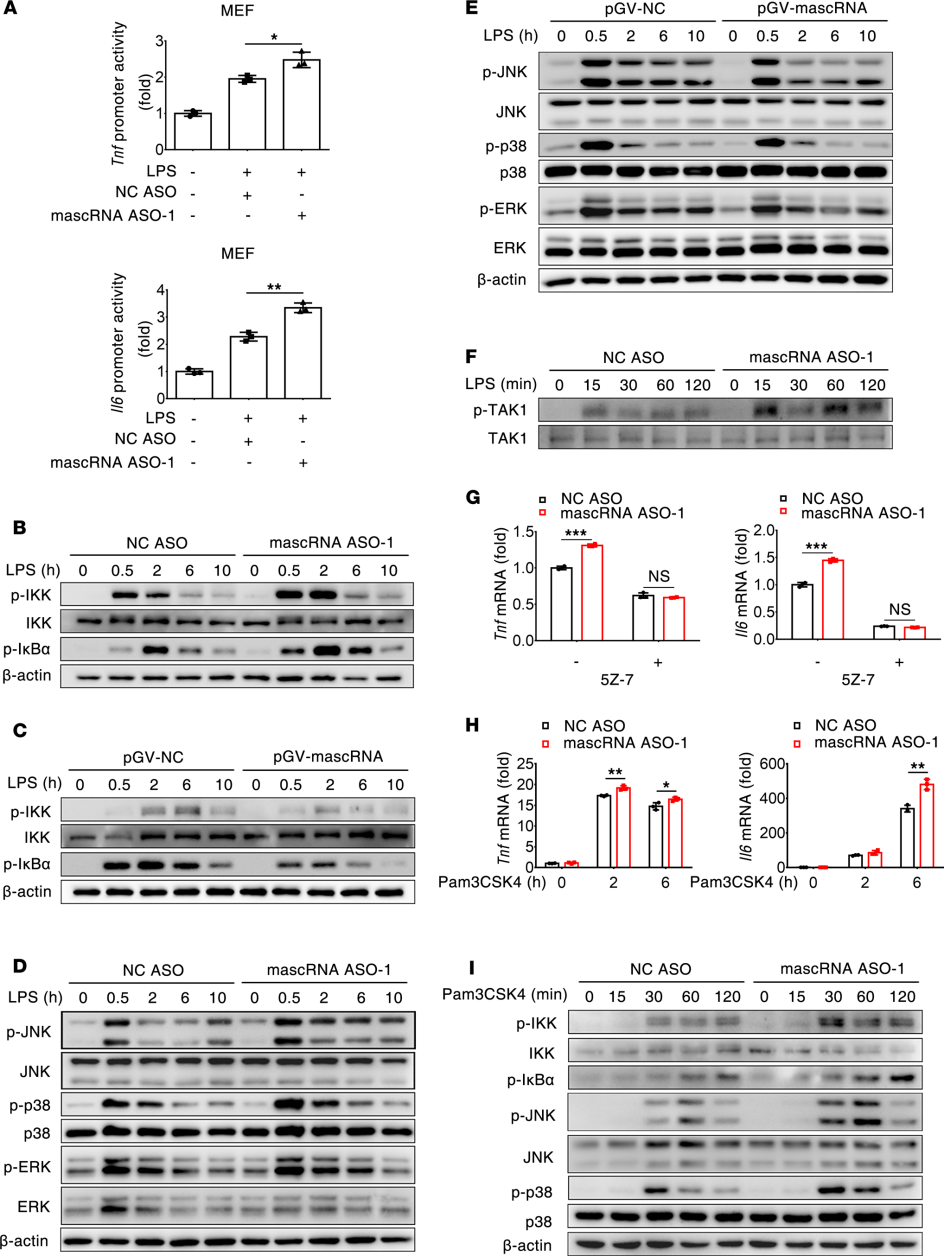
mascRNA suppresses proinflammatory cytokine transcription through inhibiting MyD88/TAK1-dependent NF-κB and MAPK signaling. (**A**) mascRNA enhances *Tnf* and *Il6* promoter activity. MEFs were transfected with mascRNA ASO or NC ASO. Twelve hours later, cells were again transfected with a reporter plasmid containing *Tnf* or *Il6* promoter–driven luciferase reporter and incubated for 48 hours. Cells were then stimulated with LPS for 6 hours. Firefly luciferase activity in cell lysates was analyzed and normalized to the activity of a cotransfected Renilla luciferase plasmid. (**B**–**E**) Effects of knockdown or overexpression of mascRNA on NF-κB and MAPK signaling in RAW264.7 cells. Cells were transfected with either mascRNA ASO (**B** and **D**) or pGV-mascRNA (**C** and **E**), stimulated with LPS for the indicated times, and then harvested for immunoblot analysis. (**F**) Immunoblot analysis of phosphorylated TAK1 (p-TAK1) and total TAK1 in mascRNA-knockdown RAW264.7 cells treated with LPS. (**G**) mascRNA inhibits TAK1 activity to suppress proinflammatory cytokine expression. mascRNA-knockdown RAW264.7 cells were pretreated with or without TAK1 kinase inhibitor 5Z-7-oxozeaenol (5Z-7) for 30 minutes prior to 2-hour (*Tnf*) or 6-hour (*Il6*) LPS challenge, followed by qPCR analysis of *Tnf* and *Il6* expression. (**H**) qPCR analysis of *Tnf* and *Il6* expression in mascRNA-knockdown RAW264.7 cells stimulated with Pam3CSK4. (**I**) Immunoblot analysis of key molecules in the NF-κB and MAPK signaling pathway in mascRNA-knockdown RAW264.7 cells stimulated with Pam3CSK4. NC, negative control. Data shown in **A**, **G**, and** H** are mean ± SD of triplicate wells. *P < 0.05; **P < 0.01; ***P < 0.001 (2-tailed Student’s *t* test). Data are representatives of 2 (**A**, **F**, and **G**) or 3 (**B**–**E**) independent experiments.

LPS-triggered production of inflammatory cytokines requires the activation of NF-κB and MAPK signaling pathways. Therefore, although there may be other explanations for mascRNA-mediated suppression of TNF and IL-6 expression, the most plausible explanation might be that mascRNA suppresses NF-κB and MAPK signaling, resulting in transcriptional repression of these cytokines. Indeed, compared with the control, mascRNA knockdown in LPS-treated mouse embryonic fibroblasts (MEFs) led to a higher level of luciferase reporter activity driven by either the *Tnf* or *Il6* promoter (Figure 2A), indicating that — unlike other small noncoding RNA (e.g., microRNA) — mascRNA exerts its regulatory effect on *Tnf* and *Il6* expression at the transcriptional level.

We next determined the effect of mascRNA on NF-κB and MAPK signaling. Compared with the control, IKK phosphorylation was enhanced in mascRNA-depleted RAW264.7 macrophages after LPS treatment (Figure 2B). Consistently, IκBα phosphorylation was more robust and remained high for a sustained period in mascRNA-depleted RAW264.7 cells compared with the control (Figure 2B). By contrast, overexpression of mascRNA ameliorated LPS-induced phosphorylation of IKK and IκBα in RAW264.7 macrophages (Figure 2C). To determine if the effect of mascRNA was solely restricted to NF-κB signaling, we also assessed the activity of MAPK signaling. We found that knockdown of mascRNA potentiated LPS-induced phosphorylation of JNK and p38 in RAW264.7 macrophages, whereas overexpression of mascRNA reduced phosphorylation levels of those proteins (Figure 2, D and E). Similarly, IκBα and JNK phosphorylation was enhanced in mascRNA-depleted MPMs (Supplemental Figure 2A). The repressive effect of mascRNA on NF-κB and MAPK signaling was further confirmed in LPS-stimulated THP-1 macrophages, as evidenced by increased phosphorylation of IKK, IκBα, and p65; increased p65 nuclear translocation; and increased phosphorylation of JNK when mascRNA was knocked down (Supplemental Figure 2, B and C).

In LPS-triggered signaling cascades, the MAPKK TAK1 acts upstream of NF-κB and MAPK and is essential for proinflammatory cytokine production. Indeed, phosphorylation of TAK1 apparently increased in mascRNA-knockdown RAW264.7 cells after LPS treatment (Figure 2F). We further determined the functional relevance of TAK1 in the regulation of mascRNA-mediated proinflammatory cytokine transcription. We found that blocking TAK1 activity with its specific inhibitor (5Z-7-oxozeaenol) abolished the increased transcription of *Tnf* and *Il6* in mascRNA-depleted RAW264.7 cells after LPS treatment (Figure 2G). Similar results were obtained in MPMs in which mascRNA was knocked down (Supplemental Figure 2D). Based on these data, we conclude that mascRNA inhibits LPS-induced NF-κB and MAPK signaling, as well as proinflammatory cytokine transcription, via suppression of TAK1.

TLR signaling can occur via 2 distinct pathways depending on the requirement of 2 different adaptor proteins, MyD88 and TRIF (1, 2). LPS is a TLR4 ligand that activates both MyD88- and TRIF-dependent pathways. To explore which signaling pathway contributes to the suppression by mascRNA, we examined TLR2 and TLR3, which signal exclusively through MyD88 and TRIF, respectively. We found that, in response to Pam3CSK4, a TLR2 ligand, there was higher expression of *Tnf* and *Il6* and stronger phosphorylation of IKK, IκBα, JNK, and p38 in mascRNA-depleted RAW264.7 macrophages than in control cells (Figure 2, H and I). Similarly, depletion of mascRNA in THP-1 macrophages promoted the production of TNF and enhanced the activation of IKK and IκBα after stimulation with Pam3CSK4 (Supplemental Figure 2E). However, there was no significant difference between mascRNA-depleted RAW264.7 cells and control cells in the expression of *Tnf* and *Il6* after stimulation with poly(I:C), a TLR3 ligand (Supplemental Figure 2F). Taken together, our data suggest that mascRNA suppresses LPS-induced proinflammatory cytokine transcription through inhibiting MyD88/TAK1-dependent NF-κB and MAPK signaling.

### mascRNA attenuates TLR4 signaling by promoting proteasomal degradation of TRAF6.

**Figure 3:**
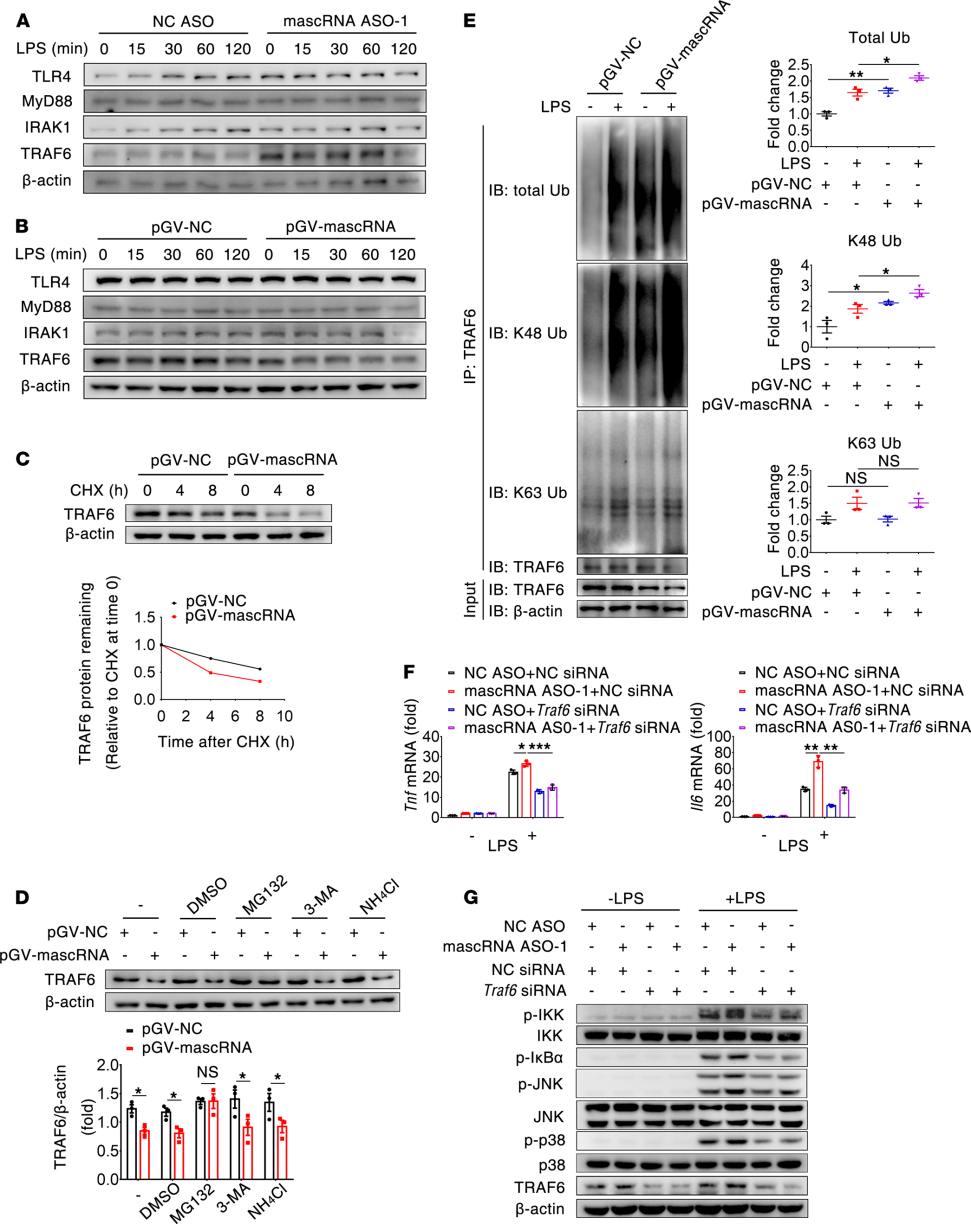
mascRNA attenuates TLR4 signaling by promoting degradation and K48-linked ubiquitination of TRAF6. (**A** and **B**) Immunoblot analysis of TLR4, MyD88, IRAK1, and TRAF6 protein abundance in mascRNA knockdown (**A**) and mascRNA-overexpressing (**B**) RAW264.7 cells stimulated with LPS for the indicated times. (**C**) mascRNA promotes TRAF6 protein degradation. mascRNA-overexpressing RAW264.7 cells were treated with 40 ng/mL cycloheximide (CHX) for the indicated times, followed by immunoblot analysis. (**D**) mascRNA promotes TRAF6 protein degradation via ubiquitin-proteasome pathway. mascRNA-overexpressing RAW264.7 cells were treated for 8 hours with 10 μM MG132, 10 mM 3-MA, 20 mM NH_4_Cl, or DMSO, followed by immunoblot analysis. TRAF6 levels were normalized to β-actin and quantified (*n* = 3 independent experiments, mean ± SEM). (**E**) mascRNA increases K48-linked ubiquitination of TRAF6. mascRNA-overexpressing or control RAW264.7 cells were stimulated with LPS for 1 hour, immunoprecipitated with an anti-TRAF6 antibody, and followed by immunoblot analysis with anti-ubiquitin, anti–K48-linked ubiquitin, anti–K63-linked ubiquitin, or anti-TRAF6. Bottom, immunoblot analysis of TRAF6 and β-actin in lysates without immunoprecipitation. Ubiquitination levels were normalized to β-actin and quantified (*n* = 3 independent experiments, mean ± SEM). (**F**) mascRNA inhibits *Tnf* and *Il6* expression via downregulating TRAF6. RAW264.7 cells were cotransfected with NC siRNA, TRAF6 siRNA, NC ASO, and mascRNA ASO in combination as indicated. Thirty-six hours after transfection, cells were stimulated with LPS for 2 (*Tnf* mRNA) or 6 (*Il6* mRNA) hours, followed by qPCR analysis. Data are mean ± SD of triplicate wells. (**G**) mascRNA suppresses LPS-induced NF-κB and MAPK signaling via downregulating TRAF6. RAW264.7 cells were transfected as in **F** and stimulated with LPS for 30 minutes, followed by immunoblot analysis. NC, negative control. *P < 0.05; **P < 0.01; ***P < 0.001 (2-tailed Student’s *t* test). Data shown in **A**–**C**, **F**, and **G** are representatives of 2 independent experiments.

Having observed that mascRNA suppresses LPS-induced TAK1 activity, we next sought to determine whether the regulatory effect of mascRNA on the inflammatory immune response was due to its influence on molecules upstream of TAK1 in the TLR4 signaling pathway, including TLR4, MyD88, IRAK1, and TRAF6. We found that knockdown or overexpression of mascRNA had no significant effects on the protein expression of TLR4, MyD88, and IRAK1; however, expression of TRAF6, an adaptor protein required for MyD88-dependent signaling, was changed. Knockdown of mascRNA significantly increased TRAF6 protein level in RAW264.7 cells with or without LPS stimulation, and overexpression of mascRNA had an opposite effect (Figure 3, A and B). Similarly, knockdown of mascRNA increased TRAF6 protein level in THP-1 macrophages with or without LPS stimulation (Supplemental Figure 3A). These results indicate that mascRNA may interfere with TLR4 signaling via reducing TRAF6 protein abundance.

The abundance of a given protein is predominantly controlled at the level of transcription and protein stability. The *TRAF6* mRNA abundance failed to change upon mascRNA overexpression in RAW264.7 cells or upon mascRNA knockdown in THP-1 macrophages (Supplemental Figure 3, B and C). Strikingly, however, treatment with the protein synthesis inhibitor cycloheximide (CHX) led to a reduced stability of TRAF6 protein in mascRNA-overexpressing RAW264.7 cells and an enhanced stability of TRAF6 protein in mascRNA-depleted THP-1 macrophages (Figure 3C and Supplemental Figure 3D). Therefore, downregulation of TRAF6 by mascRNA occurred at the level of protein, not at the level of mRNA. To investigate which degradative system dominantly regulates mascRNA-mediated degradation of TRAF6, we checked TRAF6 protein stability in the presence of specific inhibitors. We observed that mascRNA-mediated destabilization of TRAF6 was abolished by the proteasome inhibitor MG132, but not by the autophagic inhibitor 3-methyladenine (3-MA) or the lysosomal inhibitor NH_4_Cl (Figure 3D), implying that accelerated degradation of TRAF6 protein by mascRNA was dependent on the ubiquitin-proteasome pathway. Proteasomal degradation of cellular proteins typically associates with the K48-linked polyubiquitination. We therefore determined the influence of mascRNA on TRAF6 ubiquitination. As shown in Figure 3E, LPS markedly induced TRAF6 ubiquitination in RAW264.7 cells, and enforced expression of mascRNA significantly increased total and K48-linked, but not K63-linked, TRAF6 ubiquitination both before and after LPS treatment. These data indicate that mascRNA facilitates K48-linked ubiquitination of TRAF6, thus promoting its degradation by the proteasomal machinery and consequently attenuating TLR4 signaling. To further verify that mascRNA interferes with TLR4 signaling via TRAF6, we knocked down mascRNA, TRAF6, or both and found that upregulation of *Tnf* and *Il6* by mascRNA depletion was completely reversed by concomitant depletion of TRAF6 in LPS-treated RAW264.7 cells (Figure 3F). Consistent with this observation, depletion of TRAF6 abolished the increased phosphorylation of IKK, IκBα, JNK, and p38 observed in mascRNA-knockdown cells after LPS treatment (Figure 3G). Notably, a stimulatory effect of mascRNA knockdown on inflammatory signaling could still be seen in *Traf6*-depleted cells; however, this effect is significantly less strong. For example, mascRNA/*Traf6*–double knockdown markedly decreased NF-κB and MAPK activation compared with mascRNA single knockdown, whereas it only slightly increased NF-κB and MAPK activation compared with *Traf6* single knockdown (Figure 3G). Therefore, although we cannot rule out the possibility that mascRNA also functions in a TRAF6-independent manner, the suppressive effect of mascRNA on TLR4-triggered inflammatory response is mediated, at least in part, via TRAF6.

### mascRNA alleviates LPS-induced lung inflammation.

**Figure 4:**
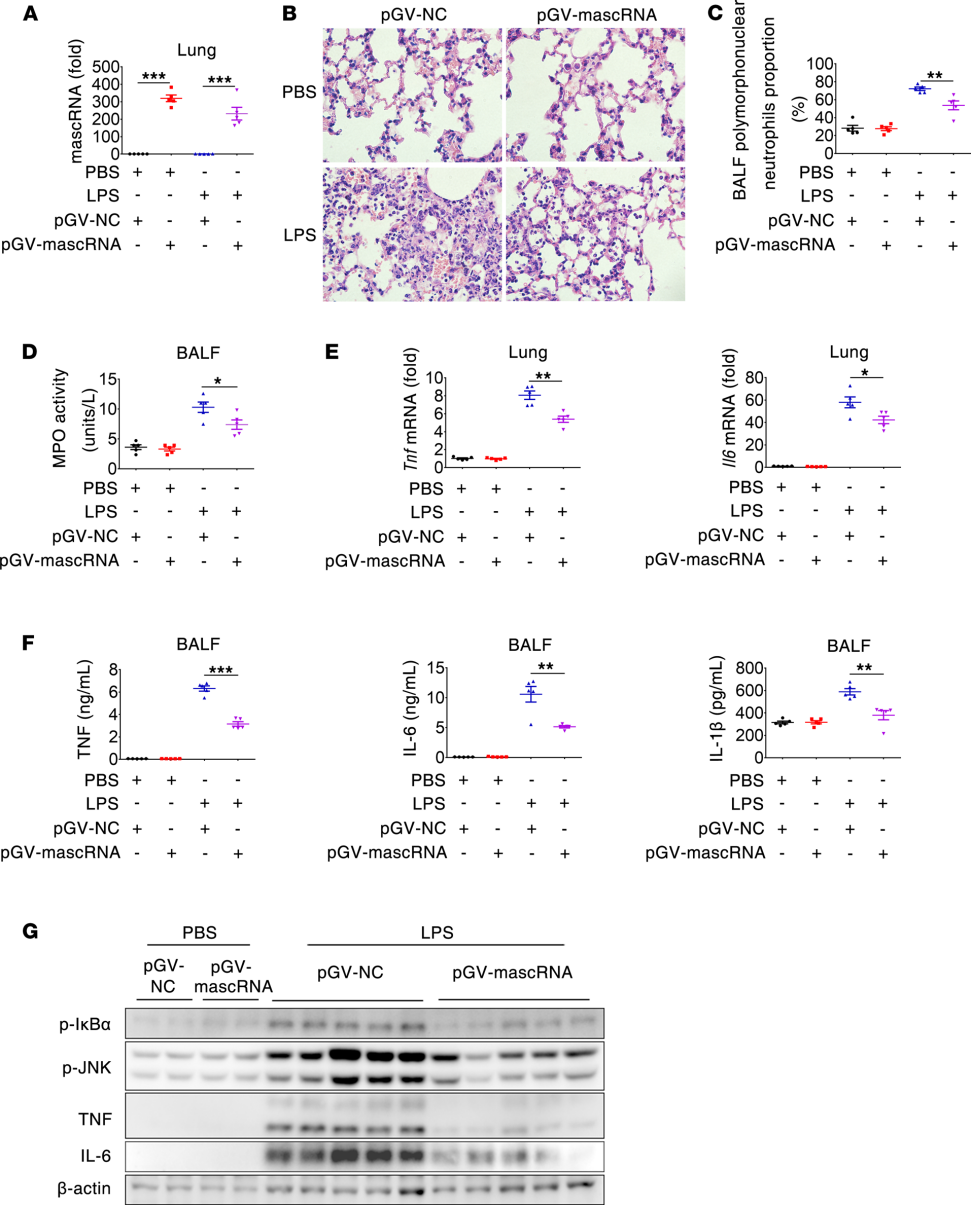
mascRNA alleviates LPS-induced lung inflammation. Mice (*n* = 5 per group) were treated oropharyngeally with either pGV-NC or pGV-mascRNA complexed with in vivo jetPEI; 48 hours after treatment, mice were challenged oropharyngeally with PBS or LPS (1 mg/kg). Six hours later, mice were sacrificed and subjected to functional analysis. (**A**) mascRNA abundance in the lungs determined by qPCR. (**B**) Representative histopathologies of lung sections stained with H&E (*n* = 5 per group). Original magnification, ×400. (**C**) Neutrophils measured in the BALFs and expressed as percentage of the total number of cells. (**D**) MPO activity in the BALFs. (**E**) *Tnf* and *Il6* mRNA abundance in the lungs determined by qPCR. (**F**) Concentration of indicated cytokines in the BALFs. (**G**) Immunoblot analysis of p-IκBα, p-JNK, TNF, and IL-6 in the lungs. Two randomly selected mice in each group challenged with PBS, and all 5 mice in each group challenged with LPS were analyzed. NC, negative control. Data shown in **A **and **C**–**F** are presented as mean ± SEM of 5 biological replicates. *P < 0.05; **P < 0.01; ***P < 0.001 (2-tailed Student’s *t* test).

Having shown that mascRNA inhibited MyD88/TRAF6/TAK1-dependent TLR signaling and downstream proinflammatory cytokine production in vitro, we next asked if mascRNA plays an immune-regulatory role in vivo. We used a mouse model of acute lung inflammation and injury induced by local administration of LPS (22). Mice were oropharyngeally instilled with a mascRNA-expressing plasmid delivered by in vivo jetPEI and, 48 hours later, treated with or without oropharyngeal LPS for 6 hours. Histological examination revealed that, compared with the control, the lungs overexpressing mascRNA displayed less severe alveolar damage in response to LPS challenge, as evidenced by decreased inflammatory cell infiltration, decreased interstitial edema, and decreased debris in the air space (Figure 4, A and B). The alleviated pulmonary inflammation in the mascRNA-overexpressing lungs was further confirmed by the observation of markedly reduced neutrophils and myeloperoxidase (MPO) activity in the bronchoalveolar lavage fluids (BALFs) (Figure 4, C and D). Also, exogenously introduced mascRNA led to less mRNA and cellular protein expression of TNF and IL-6 in the lungs, as well as less secreted TNF, IL-6, and IL-1β in BALFs (Figure 4, E–G). Consistently, exogenously introduced mascRNA suppressed LPS-induced NF-κB and MAPK signaling, as exemplified by a decrease in phosphorylation of IκBα and JNK (Figure 4G). Together, these results indicate that exogenous mascRNA mitigated LPS-induced acute lung inflammation, which was closely correlated with its suppression of inflammatory signaling and cytokine production.

### mascRNA promotes poly(I:C)-induced antiviral response.

**Figure 5:**
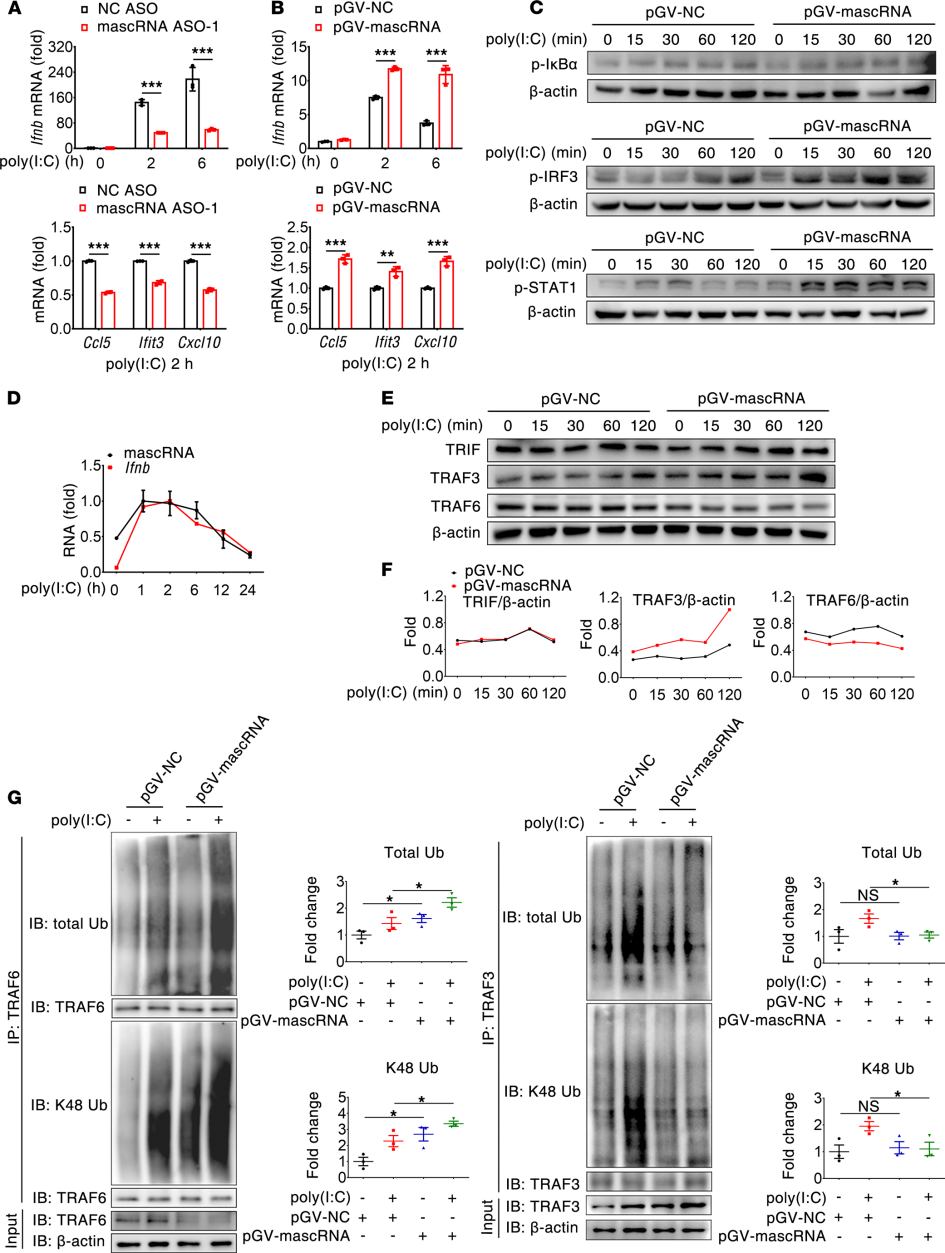
mascRNA potentiates TLR3 signaling by promoting TRAF6 degradation. (**A **and** B**) qPCR analysis of *Ifnb* and IFN-induced gene expression in mascRNA-knockdown (**A**) or mascRNA-overexpressing RAW264.7 macrophages (**B**). Cells were transfected with mascRNA ASO or mascRNA-expressing vector and then stimulated with poly(I:C) for the indicated times. (**C**) mascRNA promotes poly(I:C)-triggered TRIF/TRAF3 signaling. mascRNA-overexpressing RAW264.7 cells were stimulated with poly(I:C) for the indicated times, followed by immunoblot analysis. (**D**) Kinetics of mascRNA and *Ifnb* mRNA abundance in poly(I:C)-stimulated RAW264.7 cells. RNA was quantified by qPCR. (**E**) mascRNA decreases TRAF6 while increases TRAF3 protein abundance upon poly(I:C) stimulation. RAW264.7 cells transfected with pGV-mascRNA were stimulated with poly(I:C), followed by immunoblot analysis. (**F**) Quantitative comparison of protein expression between mascRNA-overexpressing and control cells by density scanning of the blots in **E**. (**G**) mascRNA increases poly(I:C)-induced K48-linked ubiquitination of TRAF6, while it decreases K48-linked ubiquitination of TRAF3. mascRNA-overexpressing or control cells were stimulated with poly(I:C) for 15 minutes and immunoprecipitated with an anti-TRAF6 or anti-TRAF3 antibody, followed by immunoblot analysis with anti-ubiquitin, anti–K48-linked ubiquitin, anti-TRAF6, or anti-TRAF3. Immunoblot analysis of TRAF3, TRAF6, and β-actin in lysates without immunoprecipitation. Ubiquitination levels were normalized to β-actin and quantified (*n* = 3 independent experiments, mean ± SEM). NC, negative control. Data shown in **A**, **B**, and **D** are mean ± SD of triplicate wells. *P < 0.05; **P < 0.01; ***P < 0.001 (2-tailed Student’s *t* test). Data shown in **A**–**F** are representatives of 2 independent experiments.

Having demonstrated its antiinflammatory activity, we next evaluated if mascRNA also plays a role in the antiviral immune response. We found that, in response to the TLR4 ligand LPS, mascRNA-depleted and control macrophages did not exhibit appreciable difference in the expression of *Ifnb* mRNA; similarly, mascRNA-overexpressing and control macrophages did not exhibit an appreciable difference in the phosphorylation of IRF3 and STAT1, two transcription factors required for inducing the expression of type I IFN genes and IFN-stimulated genes, respectively (Supplemental Figure 4, A and B). However, in response to the TLR3 agonist poly(I:C), a potent inducer of antiviral immune responses, mascRNA overexpression enhanced — while its knockdown inhibited — transcription of *Ifnb* and its downstream target genes, including *Ccl5*, *Ifit3,* and *Cxcl10*, in RAW264.7 cells (Figure 5, A and B). In addition, overexpression of mascRNA resulted in elevated and sustained phosphorylation of IRF3 and STAT1 triggered by poly(I:C) (Figure 5C). Notably, mascRNA did not affect poly(I:C)-induced IκBα phosphorylation, which is in agreement with the observation that mascRNA had no effect on poly(I:C)-induced *Tnf* and *Il6* transcription (Figure 5C and Supplemental Figure 2F). Collectively, these results suggest that mascRNA acts as a positive regulator of poly(I:C)-induced antiviral immune responses. In support of this view, kinetic analysis revealed a similar trend in the expression of mascRNA and *Ifnb* in poly(I:C)-treated RAW264.7 cells (Figure 5D).

**Figure 6:**
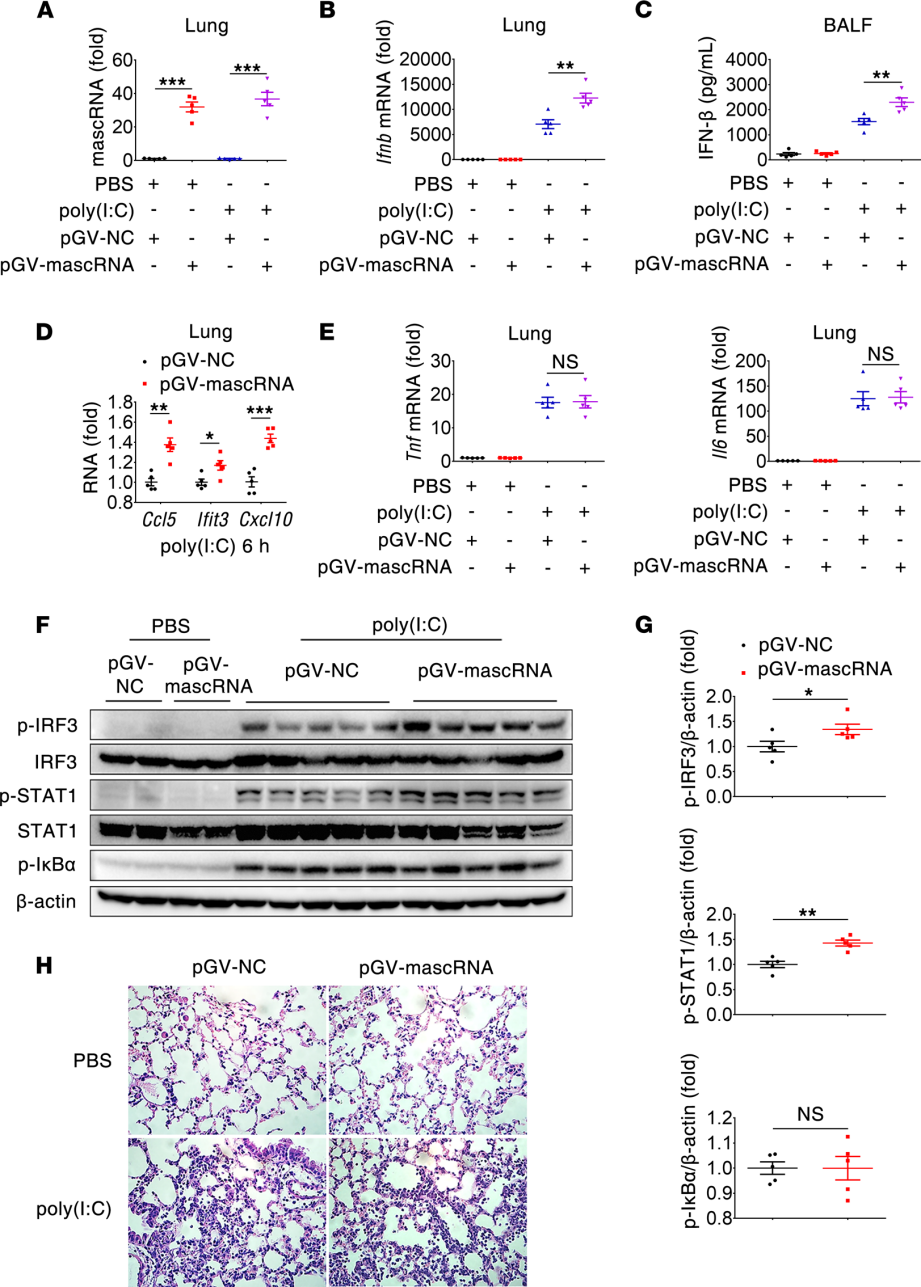
mascRNA promotes poly(I:C)-induced antiviral but not inflammatory response in vivo. Mice (*n* = 5 per group) were treated oropharyngeally with either pGV-NC or pGV-mascRNA complexed with in vivo jetPEI. Forty-eight hours after treatment, mice were challenged oropharyngeally with PBS or poly(I:C) (5 mg/kg). Six hours later, mice were sacrificed and subjected to functional analysis. (**A**) mascRNA abundance in the lungs determined by qPCR. (**B**) *Ifnb* mRNA expression in the lungs determined by qPCR. (**C**) IFN-β concentration in BALFs. (**D**) Expression of IFN-stimulated genes *Ccl5*, *Ifit3*, and *Cxcl10* in the lungs determined by qPCR. (**E**) Expression of *Tnf* and *Il6* in the lungs determined by qPCR. (**F** and **G**) Two randomly selected mice in each group challenged with PBS and all 5 mice in each group challenged with poly(I:C) were analyzed. Indicated proteins in the lungs were assessed by immunoblot (**F**), and fold change of phosphorylated proteins was normalized to β-actin and quantified by densitometric scanning of blots (**G**). (**H**) Representative histopathologies of lung sections stained with H&E (*n* = 5 per group). Original magnification, ×400. NC, negative control. Data shown in **A**–**E** and **G** are mean ± SEM of 5 biological replicates. *P < 0.05; **P < 0.01; ***P < 0.001 (2-tailed Student’s *t* test).

The role of mascRNA in regulating IFN antiviral responses was further assessed in vivo using a mouse model of lung inflammation induced by poly(I:C). Compared with the control, enforced expression of mascRNA led to increased expression of *Ifnb* and IFN-stimulated genes (e.g., *Ccl5*, *Ifit3* and *Cxcl10*) in lung tissues, together with increased IFN-β secretion in BALF (Figure 6, A–D), whereas expression of *Tnf* and *Il6* did not differ significantly between the 2 groups (Figure 6E). Consistently, exogenous mascRNA enhanced poly(I:C)-induced IRF3 and STAT1 phosphorylation, but it had no impact on IκB phosphorylation in the lung tissues (Figure 6, F and G). Additionally, histological analysis revealed no difference in the severity of poly(I:C)-induced lung inflammation between control and mascRNA-overexpressing plasmid–treated mice (Figure 6H). Hence, mascRNA had a major effect on TLR3-triggered antiviral but not proinflammatory response both in vitro and in vivo.

Poly(I:C)-induced antiviral immunity depends critically on TRIF/TRAF3 pathway to initiate type I IFN production. We therefore assessed key signaling molecules in this pathway. Western blot analysis revealed that overexpression of mascRNA did not have a significant effect on TRIF protein abundance in poly(I:C)-treated RAW264.7 cells, but the amount of TRAF3 protein was increased at all time points examined (Figure 5, E and F). Interestingly, protein expression of TRAF3 inversely correlated with that of TRAF6 whose amount was decreased at all time points in poly(I:C)-treated mascRNA-overexpressing cells (Figure 5, E and F). Since knockdown or overexpression of mascRNA did not affect poly(I:C)-induced transcription of *Traf3* (Supplemental Figure 4, C and D) and TRAF6 is required for LPS-induced degradative ubiquitation of TRAF3 (23), we reasoned that, in response to poly(I:C), mascRNA may promote TRAF6 degradation, which in turn resulted in inhibition of TRAF3 degradation. To substantiate this hypothesis, we examined the effect of mascRNA on the ubiquitination of TRAF6 and TRAF3 in poly(I:C)-treated RAW264.7 cells. Poly(I:C) enhanced ubiquitination of TRAF6, and importantly, overexpression of mascRNA resulted in more poly(I:C)-induced K48-linked ubiquitation of TRAF6 but less poly(I:C)-induced K48-linked ubiquitation of TRAF3 (Figure 5G). Moreover, poly(I:C)-induced K48-linked ubiquitation of TRAF3 was TRAF6 dependent (Supplemental Figure 4E). Hence, mascRNA promotes degradative ubiquitation of TRAF6 to differentially regulate MyD88- and TRIF-dependent TLR signaling pathways.

### mascRNA interacts with hnRNP H/F.

**Figure 7:**
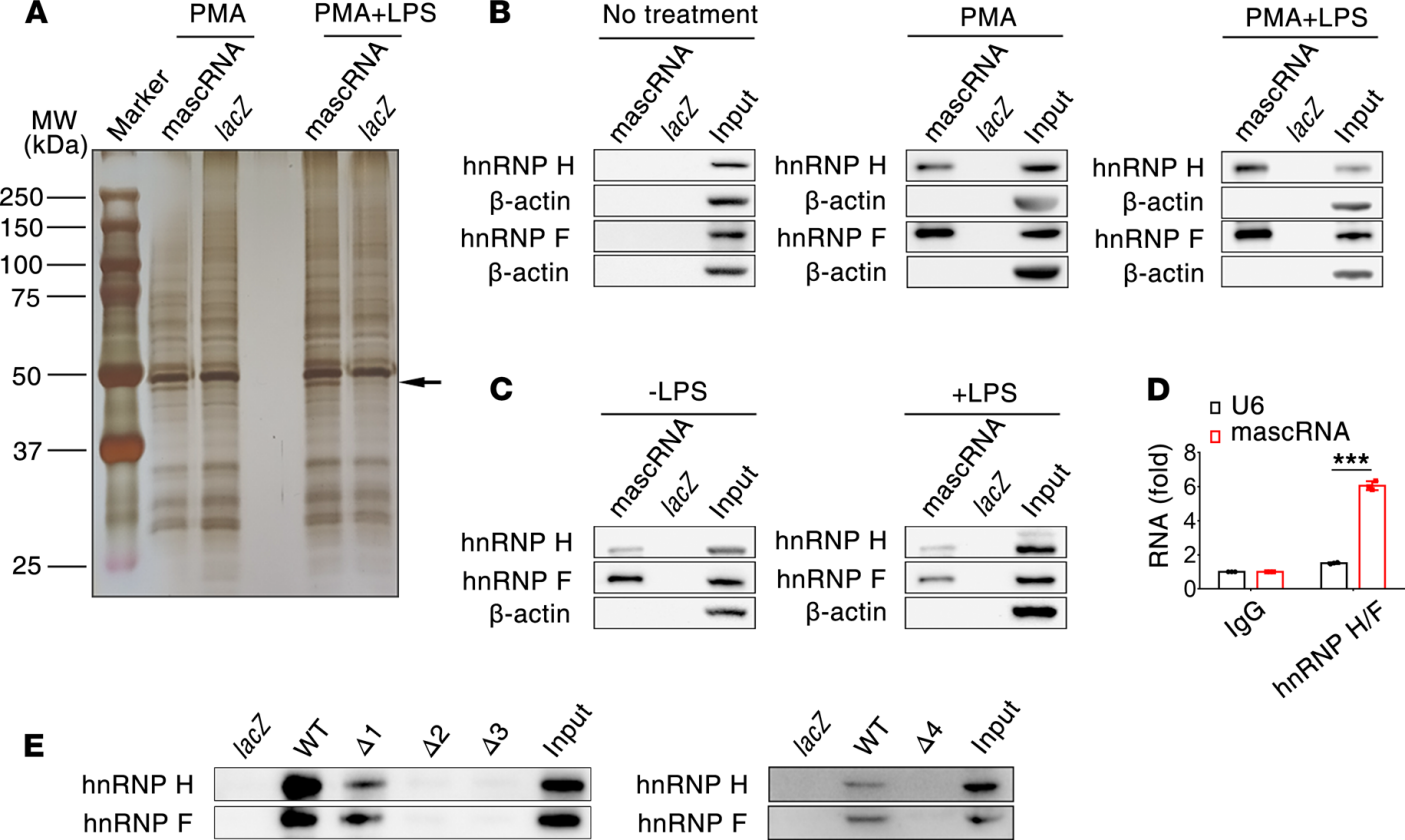
mascRNA interacts with hnRNP H/F. (**A**) Silver staining of mascRNA-associated proteins in PMA-differentiated THP-1 cytoplasmic lysates. The bound proteins were pulled down using biotinylated mascRNA and identified by mass spectrometry–based quantitative proteomics. Arrow indicates specific proteins pulled down by mascRNA. (**B**) Immunoblot analysis of proteins pulled down as in **A** from THP-1 monocytes, PMA-differentiated THP-1 macrophages, and THP-1 macrophages treated with LPS for 2 hours. (**C**) Immunoblot analysis of proteins pulled down as in **A** from cytoplasmic lysates of RAW264.7 cells treated with or without LPS for 2 hours. (**D**) Total cell lysates of RAW264.7 cells treated with LPS for 2 hours were immunoprecipitated with mouse IgG or hnRNP H/F antibody, and the abundance of mascRNA and U6 RNA in the precipitates was analyzed by qPCR. (**E**) Immunoblot of hnRNP H and F in the samples pulled down by biotinylated WT mascRNA and its 4 deletion mutants from cytoplasmic lysates of THP-1 macrophages. A 61 nt *lacZ* mRNA fragment was used as negative control. Data shown in **D** are the mean ± SD of triplicate wells. ***P < 0.001 (2-tailed Student’s *t* test). Data shown in **A**–**D** are representatives of 2 independent experiments.

Many noncoding RNA molecules play their role by physically interacting with other proteins. To understand how mascRNA suppresses TLR signaling-mediated proinflammatory cytokine transcription, we sought to identify potential cellular proteins associated with mascRNA using RNA pull-down assay. As mascRNA was mainly localized in the cytoplasm of macrophages (Supplemental Figure 5A), biotinylated mascRNA was incubated with cytoplasmic lysates of resting or LPS-activated THP-1 macrophages and pulled down using streptavidin beads. The proteins retrieved were resolved by SDS-PAGE, and a band of ~49 kDa from both resting and activated THP-1 macrophages was specifically pulled down by mascRNA but not by an unrelated *lacZ* mRNA segment with similar size (Figure 7A). Quantitative mass spectrometry analysis revealed 2 mascRNA-binding proteins, hnRNP H (hnRNP H1) and F, both of which were significantly enriched in the pull-down products of mascRNA but absent from that of *lacZ* mRNA control. Notably, the size of hnRNP H (449 amino acids) is in agreement with the molecular weight of the band specific to mascRNA in the protein gel. We repeated the pull-down assay, and the interaction between mascRNA and hnRNP H/F in resting as well as LPS-activated THP-1 macrophages was confirmed by Western blot analysis; however, this interaction was not detectable in THP-1 monocytes (Figure 7B), suggesting that mascRNA-hnRNP H/F interaction may be specific to cell type. Likewise, mascRNA pull-down also recovered hnRNP H/F from cytoplasmic lysates of resting and LPS-stimulated RAW264.7 cells (Figure 7C). In addition, an antibody against hnRNP H/F was used to immunoprecipitate endogenous hnRNP H/F from RAW264.7 cell extracts, and we observed enrichment of mascRNA, but not U6 RNA, in the anti–hnRNP H/F immunoprecipitates compared with a nonspecific antibody (Figure 7D). Based on these results, we conclude that association of mascRNA and hnRNP H/F is conserved at least in mouse and human macrophages.

We next sought to determine which part of mascRNA is responsible for its binding to hnRNP H/F. mascRNA was predicted to display a tRNA-like secondary structure composed of 3 hairpins (regions 1–3) and 1 stem bearing an unpaired CCA motif at the 3′ end (region 4) (Supplemental Figure 5B). We constructed 4 discrete deletion mutants (Δ1–Δ4), among which Δ1, Δ2, and Δ3 lack the entire hairpin structure of region 1, 2, and 3, respectively, and Δ4 deleted part of the region 4, including the 3′-terminal CCA. These deletion constructs were designed not to alter the secondary structure of the remaining mascRNA sequence as analyzed by RNA secondary structure prediction software RNAfold. The mascRNA mutants were biotin labeled and used to pull down hnRNP H/F from THP-1 macrophage extract. As shown in Figure 7E, the hnRNP H/F binding activity of mascRNA mapped to 2 hairpins in regions 2 and 3, as well as the stem in region 4.

hnRNP H/F are reported to interact directly with G-rich motifs and mascRNA contains a GGGG motif (nt 35–38) in region 3. To determine if the G-tract is involved in the interaction with hnRNP H/F, we constructed 2 point mutations, G36A and GG36-37AA (Supplemental Figure 5C). Because the 2 mutations were predicted to alter local secondary structures, we also constructed their corresponding compensatory mutations, G36A/C47T and GG36-37AA/CC46-47TT, which restored local base pairing and returned their entire secondary structure to that of the WT. We observed that biotinylated G36A, GG36-37AA, and G36A/C47T mutants — and, to a lesser extent, the GG36-37AA/CC46-47TT mutant — specifically retrieved hnRNP H/F from cytoplasmic lysates of THP-1 macrophages (Supplemental Figure 5D). Weak hnRNP H/F signals retrieved by the GG36-37AA/CC46-47TT mutant may be due to low biotin labeling efficiency. These findings indicate that the GGGG motif in mascRNA is not important for its association with hnRNP H/F.

### hnRNP H/F promote mascRNA degradation to exert a proinflammatory effect.

**Figure 8:**
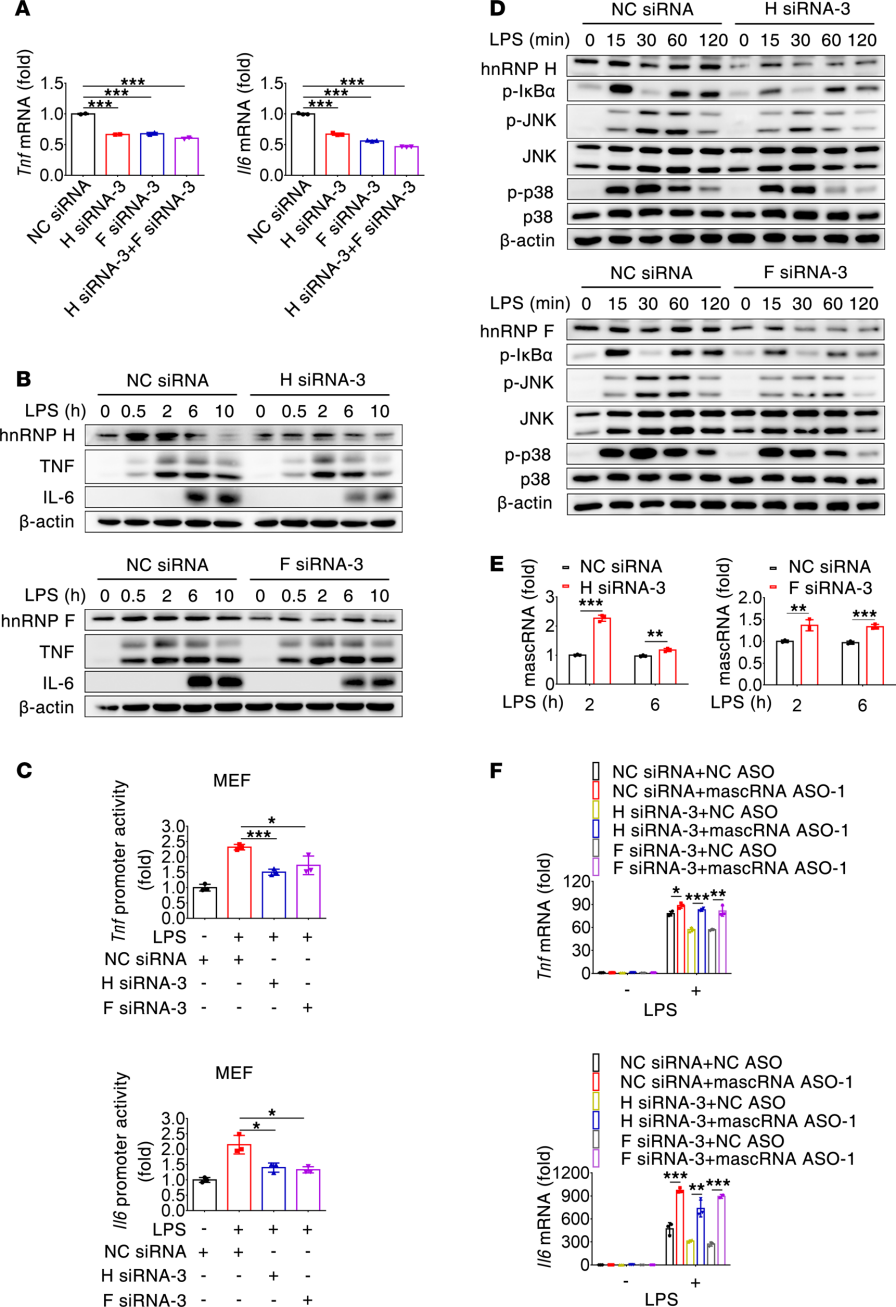
hnRNP H/F promote mascRNA degradation to exert a proinflammatory effect. (**A**) qPCR analysis of *Tnf* and *Il6* mRNA abundance in hnRNP H– or/and hnRNP F–knockdown RAW264.7 cells stimulated with LPS for 2 hours. (**B**) Immunoblot analysis of cellular TNF and IL-6 precursors in hnRNP H– or hnRNP F–knockdown RAW264.7 cells stimulated with LPS for the indicated times. (**C**) hnRNP H and F enhance *Tnf* and *Il6* promoter activity. MEFs were transfected with a *Tnf* or *Il6* promoter–luciferase reporter construct and an indicated siRNA. Forty-eight hours after transfection, cells were stimulated with LPS for 6 hours. Firefly luciferase activity was analyzed in cell lysates and normalized to the activity of a cotransfected Renilla luciferase plasmid. (**D**) Immunoblot analysis of key molecules in NF-κB and MAPK signaling pathways in hnRNP H– or hnRNP F–knockdown RAW264.7 cells stimulated with LPS for the indicated times. (**E**) qPCR analysis of mascRNA abundance in hnRNP H– or hnRNP F–knockdown RAW264.7 cells treated with LPS for the indicated times. (**F**) mascRNA mediates the role of hnRNPs H or F in the regulation of inflammatory responses. Mouse peritoneal macrophages were cotransfected with NC siRNA, *hnRNP H* siRNA, *hnRNP F* siRNA, NC ASO, or mascRNA ASO in combination as indicated. Thirty-six hours after transfection, cells were stimulated with LPS for 2 (*Tnf*) or 6 (*Il6*) hours, followed by qPCR analysis of *Tnf* and *Il6* expression. NC, negative control. Data are mean ± SD of triplicate wells (**A**, **C**, **E**, and **F**). *P < 0.05; **P < 0.01; ***P < 0.001 (2-tailed Student’s *t* test). Data are representatives of 2 (**C**, **D**, and **E**) or 3 (**A** and **B**) independent experiments.

Given the association of mascRNA with hnRNP H/F, we reasoned that hnRNP H/F might affect proinflammatory cytokine production. Expression of hnRNP H and/or hnRNP F was depleted in RAW264.7 cells using siRNA-mediated gene silencing. Three different siRNA for each gene were tested for their silencing efficiency using immunoblot, the most efficient of which (*hnRNP H* siRNA-3, *hnRNP F* siRNA-3) was selected for subsequent experiments (Supplemental Figure 6A). As shown in Figure 8A, knockdown of hnRNP H or/and F significantly decreased *Tnf* and *Il6* mRNA abundance in LPS-stimulated RAW264.7 macrophages. Immunoblot analysis also showed a decrease in cellular IL-6 protein levels upon hnRNP H or F knockdown; however, the effect of hnRNP H or F knockdown on TNF protein expression was not evident (Figure 8B). In similar assays, hnRNP H and/or F depletion resulted in reduced expression of TNF and IL-6 mRNA and protein in LPS-induced MPMs (Supplemental Figure 6, B and C). Thus, hnRNP H/F are involved in fine-tuning innate immune-mediated production of proinflammatory cytokines.

Cytokine expression in macrophages is known to be regulated at multiple levels, such as transcription, mRNA stability, and translation. To better understand the function of hnRNP H/F, we transfected MEFs with a *Tnf* or *Il6* promoter–luciferase reporter construct. Knockdown of hnRNP H or F with their respective siRNA attenuated LPS-induced luciferase activity driven by either *Tnf* or *Il6* promoter (Figure 8C), indicating that hnRNP H/F affect proinflammatory cytokine expression at least at the transcriptional level. Consistently, knockdown of hnRNP H or F compromised the phosphorylation of IκBα, JNK, and p38 in LPS-treated RAW264.7 cells (Figure 8D). Together, these data suggest that, in contrast to mascRNA, hnRNP H/F enhance NF-κB, p38, and JNK signaling to exert a positive effect on *Tnf* and *Il6* expression.

Because hnRNP H/F and mascRNA have opposing effects on inflammatory cytokine responses in macrophages upon TLR4 engagement, we then explored functional significance of the interaction between hnRNP H/F and mascRNA. Antisense knockdown of mascRNA did not seem to have much effect on hnRNP H and F protein levels in LPS-treated RAW264.7 cells (Supplemental Figure 6D). On the contrary, siRNA-mediated knockdown of hnRNP H or F significantly increased mascRNA abundance in LPS-treated RAW264.7 macrophages and MPMs (Figure 8E and Supplemental Figure 6E). Thus, binding of hnRNP H/F to mascRNA may decrease its stability, although it is still possible that hnRNP H/F impact mascRNA expression in a contact-independent manner. These data suggest that hnRNP H/F function upstream of mascRNA in the regulation of TLR4-triggered proinflammatory responses. To test if the proinflammatory effect of hnRNP H/F depend on mascRNA, we silenced hnRNP H/F, mascRNA, or both and found that mascRNA knockdown completely rescued the impaired expression of *Tnf* and *Il6* in hnRNP H/F–depleted MPMs at 2 and 6 hours, respectively, after LPS treatment (Figure 8F). Thus, mascRNA mediates the proinflammatory effect of hnRNP H/F in macrophages.

## Discussion

In the present work, we demonstrate that mascRNA negatively regulates TLR4/2-triggered MyD88-dependent NF-κB and MAPK signaling to suppress proinflammatory cytokine production, and this tRNA-like RNA has an antiinflammatory activity both in vitro and in vivo. Furthermore, our results provided a mechanism by which mascRNA exerts its antiinflammatory effects through promoting K48-linked ubiquitination and proteasomal degradation of TRAF6. TRAF6 is an E3 ubiquitin ligase that, upon ligand stimulation, can autoubiquitnate itself, generating K63-linked polyubiquitin chains essential for recruiting and activating downstream kinases (1, 4). As a key player in TLR signaling pathways, TRAF6 activity is tightly regulated through multiple mechanisms — especially at the level of posttranscription and posttranslation. The mechanisms controlling TRAF6 function include, but are not limited to, deconjugating its K63 ubiquitin chains via deubiquitinases (6), promoting its K48-linked ubiquitination by ubiquitin ligases (7, 8), preventing TRAF6 oligomerization and autoubiquitination by interacting with other proteins or lncRNAs (9, 10), and promoting *TRAF6* mRNA degradation by miRNAs (16). Given its small size and tRNA-like structure, identification of mascRNA as a negative regulator represents an additional layer of the modulatory architecture beyond proteins, lncRNAs, and microRNAs for the control of TRAF6 activity. At present, how mascRNA promotes K48-linked ubiquitination of TRAF6 remains mysterious. While this work was in progress, Lu et al. reported that mascRNA associates with glutaminyl-tRNA synthetase (QARS) in HEK293 cells and promotes global protein translation and cell proliferation by upregulating QARS protein stability (24). However, biotinylated mascRNA did not pull down QARS from the lysates of THP-1 macrophages (data not shown), and knockdown of QARS did not alter *Tnf* and *Il6* mRNA abundance in LPS-treated RAW264.7 cells (Supplemental Figure 7A). Moreover, we confirmed that knockdown of QARS did not alter TRAF6 protein abundance in RAW264.7 cells with or without LPS stimulation (Supplemental Figure 7B). These results suggest that mascRNA-mediated suppression of proinflammatory responses is independent of QARS and that mascRNA may function in a cell type–specific manner. Recently, tRFs — the fragments generated from tRNA — represent an emerging category of regulatory RNAs (25, 26). It would be interesting to determine if mascRNA acts as a tRF to regulate those factors that affect K48-linked ubiquitination of TRAF6.

In addition to negatively regulating TLR4/2-triggered inflammatory activity, mascRNA positively regulates TLR3-induced antiviral responses through promoting TRAF6 degradation, which in turn resulted in reduced degradation of TRAF3, an essential component in the TRIF-dependent pathway. While mascRNA dampened TRAF6 protein abundance, this small RNA selectively potentiated the IFN response without a discernible effect on the activation of NF-κB and the induction of proinflammatory cytokines in poly(I:C)-treated macrophages (Figure 5C and Supplemental Figure 2F), consistent with the notion that TRAF6 is dispensable for TLR3-triggered proinflammatory signaling in macrophages (27, 28). Notably, Jiang et al. reported that TRAF6 is involved in poly(I:C)-induced activation of NF-κB in MEFs (29). The reason for this discrepancy is unknown. One interpretation is that the role of TRAF6 in TLR3 signaling may be cell type–specific. Interestingly, in response to LPS, mascRNA preferentially suppressed inflammatory response with little effect on the induction of *Ifnb*. Although mascRNA enhanced TRAF3 protein expression upon LPS stimulation (Supplemental Figure 4B), our experimental system (e.g., antibodies used) is probably not sensitive enough to detect a modest increase in TLR4-triggered TRAF3-dependent antiviral response, which is much weaker than that triggered by TLR3 (30). Hence, by downregulating TRAF6 protein stability, mascRNA differentially regulates 2 major responses, one being MyD88/TRAF6-dependent proinflammatory cytokine production upon TLR4 activation and the other being TRIF/TRAF3-dependent induction of type I IFNs upon TLR3 activation. The 2 immune responses are known to have contrasting roles in the pathogenesis of some diseases. For example, type I IFNs are generally thought to activate the immune system and drive autoimmunity, yet as pleiotropic cytokines, they sometimes exert potent suppressive effects on inflammation in certain immune-mediated inflammatory disorders, including multiple sclerosis (31), ulcerative colitis (32, 33), and inflammatory arthritis (34). Furthermore, malignant cell growth and metastasis are promoted by inflammatory stimuli (e.g., TNF) but can be therapeutically inhibited by type I IFNs (35). Therefore, mascRNA holds a promise for the treatment of these diseases because it inhibits proinflammatory cytokine production without concomitantly compromising type I IFN–based suppressive effect on inflammation and tumor.

mascRNA is derived from processing of the pre-Malat1 transcript, which generates not only mascRNA, but also mature Malat1 (17). In this sense, Malat1 and mascRNA are subject to the same transcriptional regulation. Consistently, both mascRNA (Figure 5D) and Malat1 (36) reached a maximum expression peak at 1 hour and then started to decrease 3–6 hours after poly(I:C) stimulation. However, the 2 genetically related noncoding RNAs did not show similar expression profiles in LPS-treated macrophages: expression of Malat1 increased (19, 36), whereas that of mascRNA quickly decreased, within 2 hours after LPS stimulation (Figure 1A). The decreased mascRNA expression at an early stage during LPS treatment may be attributed to an undefined posttranscriptional regulatory mechanism which, for instance, influences mascRNA stability. It is worth noting that mascRNA not only affected TRAF6 protein expression in LPS or poly(I:C)-treated macrophages, but also markedly decreased basal TRAF6 protein expression in resting macrophages (Figure 3, A and B, and Supplemental Figure 3A). Based on the phenomena observed in this work and the high abundance of mascRNA in immune cells (21), we propose that, by lowering TRAF6 abundance, mascRNA serves as a brake on TLR/MyD88 signaling to prevent induction of inflammatory responses in resting macrophages. Upon stimulation by a TLR ligand that activates MyD88 signaling, mascRNA expression abruptly declines (possibly through a posttranscriptional mechanism) to allow the production of proinflammatory cytokines; later, mascRNA expression increases (most likely through TLR-triggered transcriptional activation of Malat1 precursor) to prevent uncontrolled inflammation from spreading. On the contrary, in response to a TLR ligand that activates TRIF signaling, mascRNA is upregulated and serves as an accelerator on TRIF-dependent induction of type I IFNs. Since TLR4 signals through both MyD88 and TRIF, while TLR3 signals only through TRIF, mascRNA downregulation by TLR4 and upregulation by TLR3 suggest that MyD88 signaling may lead to strong downregulation of mascRNA, which could not be overcome by the TRIF-dependent upregulation of mascRNA. Indeed, Pam3CSK4, a ligand of TLR2 that signals only through MyD88, induced further downregulation of mascRNA compared with LPS (Supplemental Figure 8).

Previous studies showed that Malat1 also negatively regulates LPS-induced inflammatory cytokine expression (19, 20). We reason that mascRNA functions independently of Malat1 in attenuating inflammation because (a) they are localized in different cell compartments (17), (b) depletion of mascRNA did not affect Malat1 abundance (Supplemental Figure 1A), and (c) Malat1 associates with NF-κB in the nucleus while mascRNA promotes TRAF6 degradation in the cytoplasm, so they differ in their mechanism of action (19). Nonetheless, it is still possible that mascRNA contributes to the antiinflammatory activity of Malat1 because knockdown or deletion of Malat1 in these studies may impair the generation of functional mascRNA (19, 20). In addition, it needs to be pointed out that, in sharp contrast to mascRNA, work by Liu et al. revealed a suppressive effect of Malat1 on antiviral immunity (36). This discrepancy could be explained by the observation that Liu et al. used virus rather than poly(I:C) to trigger antiviral immunity. In fact, expression of Malat1 is decreased upon viral infection but is increased upon stimulation with TLR ligands such as LPS and poly(I:C) (36). Hence, it would be interesting to investigate whether and how mascRNA influences antiviral immunity upon viral infection.

In this study, hnRNP H/F were demonstrated to bind mascRNA and promote its degradation in macrophages. Interestingly, hnRNP H/F were among the 127 proteins identified by Chen et al. that potentially interacted with an RNA segment corresponding to the 3′ terminal region of human MALAT1 (nt 6918–8441) (37). The segment used by Chen et al. contained a full-length mascRNA sequence. Based on our data, it is likely that the region of mascRNA in the MALAT1 segment (nt 6918–8441) mediated its interaction with hnRNP H/F. By constructing a series of deletion mutants predicted not to disturb the secondary structure of the remaining sequence, we found that 2 hairpins and the CCA stem in the tRNA-like structure of mascRNA contribute to hnRNP H/F binding. The structural basis of mascRNA responsible for interacting with hnRNP H/F remains elusive. Nevertheless, 2 point mutations in the G tract (G36A, GG36-37AA) of hairpin 3 predicted to disrupt local secondary structures were still capable of binding to hnRNP H/F, indicating that it is the primary sequence of mascRNA that mediates its association with hnRNP H/F. Unlike most hnRNPs, which bind RNA and single-stranded DNA independently of sequence, hnRNP H/F are documented to be specific for 3 consecutive guanines (38). Our data indicate hnRNP H/F can bind to RNA sequences other than poly(G) tracts.

hnRNPs are ubiquitously expressed factors that regulate gene expression at different levels, including transcription, mRNA stabilization, alternative splicing, and translation. Among hnRNPs, hnRNP H/F are best known for their role in regulating alternative splicing (38). In the present work, 4 independent lines of evidence — including decreased *Tnf* and *Il6* promoter activities, decreased NF-κB and MAPK signaling, increased levels of mascRNA, and the complete rescue of impaired *Tnf* and *Il6* expression by mascRNA knockdown in hnRNP H/F–depleted LPS-treated cells — all supported the conclusion that hnRNP H/F positively affect TLR4 signaling and, thus, the transcription of *Tnf* and *Il6* through downregulating mascRNA abundance, although participation of hnRNP H/F in splicing regulation of these 2 cytokines could not be excluded. Of note, a previous study reported that global mRNA sequencing (mRNA-seq) analysis did not reveal splicing changes in *TNF* and *IL6* mRNA following hnRNP H knockdown in 293T cells, and cross-linking immunoprecipitation sequencing (CLIP-seq) analysis did not identify endogenous *TNF* and *IL6* mRNA bound by hnRNP H (39), which can serve as an additional evidence that hnRNP H/F exert a mascRNA-mediated regulatory effect on *Tnf* and *Il6* expression at the level of transcription rather than posttranscription.

In summary, our findings uncover a mechanism underlying the regulation of innate immunity by mascRNA, a tRNA-like cytoplasmic noncoding RNA whose stability is affected by its binding proteins hnRNP H/F. By promoting degradative ubiquitination of TRAF6, mascRNA negatively regulates TLR-mediated MyD88-dependent proinflammatory signaling while it positively regulates TRIF-dependent IFN signaling. The dual role of mascRNA in regulating innate immune signaling could hold promise for developing therapeutics for the treatment of certain inflammation-related diseases since mascRNA suppresses inflammatory cytokine production without compromising IFN-based therapeutic effects on inflammation and cancer.

## Methods

### Cell culture.

RAW264.7 cells (Cell Bank of Chinese Academy of Sciences, catalog SCSP-5036), MEFs (provided by Zhenyi Su, Southeast University, Nanjing, China) and BMDMs were cultured in DMEM (Thermo Fisher Scientific, catalog 11965092), whereas THP-1 cells (Cell Bank of Chinese Academy of Sciences, catalog SCSP-567) and MPMs were cultured in RPMI 1640 (Thermo Fisher Scientific, catalog 11879092). MPMs were obtained from the 6- to 8-week-old C57BL/6 male mice by i.p. injection of 2 mL of sterile 3% thioglycollate medium. Four days after injection, mice were sacrificed, and cells were harvested by a lavage of the peritoneal cavity with 10 mL of DMEM medium. After adhesion to plastic culture dishes for 2 hours, the cells were washed 3 times with phosphate buffer saline (PBS) to remove unattached cells. The adhered cells were the MPMs, which were used in further experiments. BMDMs were derived from BM cells of mice as described previously and with the following modifications (40). Briefly, following erythrocyte lysis, BM cells were flushed from the tibia and femurs of 8- to 10-week-old C57BL/6 female mice and then isolated by ficoll-hypaque density-gradient centrifugation (1000*g* for 30 minutes at room temperature). Cells were differentiated with 100 ng/mL mouse M-CSF recombinant protein (Thermo Fisher Scientific, catalog PMC 2044) for 6 days, with the replacement of culture medium every 2–3 days. The differentiated cells were then split and plated for subsequent experiments. THP-1 monocytes were differentiated into macrophages by treatment with 40 ng/mL PMA for 24 hours, and they were then cultured in fresh medium prior to further treatment. To induce a TLR-mediated response, cells were treated with LPS (100 ng/mL), Pam3CSK4 (100 ng/mL) or poly(I:C) (25 μg/mL). All the cell media were supplemented with 10% (vol/vol) of FBS (Thermo Fisher Scientific, catalog 10270-106), and 1% penicillin-streptomycin (Thermo Fisher Scientific, catalog 15140122). All the cells were grown at 37°C, 5% CO_2_ in the fully humidified air.

### Cell transfection.

For transient transfection, cells were transfected with ASO or siRNA by means of Lipofectamine-RNAi MAX (Invitrogen, catalog 13778150) or transfected with plasmid by means of Lipofectamine 2000 (Invitrogen, catalog 11668019) according to the manufacturers’ instruction. The sequences of ASO and siRNA are described in Supplemental Table 1. For stable transfection, RAW264.7 cells were transfected with pGV-mascRNA or empty vector (pGV-NC) by means of jetPEI-Macrophage (Polyplus, catalog 103-05N) according to the manufacturers’ instruction. Twenty-four hours after transfection, cells were screened by G-418 (400 ng/mL; Beyotime Biotechnology; catalog ST081) for 10 days. The stably transfected cell pools were then split and plated for subsequent experiments.

### In vitro transcription and biotin labeling.

To prepare mascRNA mutant templates for in vitro transcription, 2 overlapping oligonucleotides with desired deletion or point mutation were mixed and subjected to PCR in the presence of a specific primer pair listed in Supplemental Table 1. The forward primer contains T7 promoter sequence in addition to the sequence corresponding to the 5′ end of mascRNA, while the reverse primer was an oligo corresponding to the 3′ end of each mascRNA mutant. The PCR-amplified products were purified by phenol/chloroform extraction and subject to in vitro transcription following the manufacturers’ instruction (Takara, catalog 2540A). The transcribed RNAs were treated with RNase-free DNase I (Takara, catalog 2270A), purified by chloroform/isoamyl alcohol extraction, and labeled with biotin using the Pierce RNA 3′ End Desthiobiotinylation Kit (Thermo Fisher Scientific, catalog 20163). Biotinylation was confirmed by dot blot using a biotin detection kit (Beyotime Biotechnology, catalog D3308).

### RNA pull-down assay.

Biotin-labeled WT mascRNA and *lac*Z mRNA segment were synthesized by GenePharma. In total, 50 pmol of biotinylated RNA (WT human mascRNA: 5′-GAUGCUGGUGGUUGGCACUCCUGGUUUCCAGGACGGGGUUCAGAUCCCUGCGGCGUCUCCA-3′; negative control *lac*Z fragment: 5′-CGGAUUCACUGGCCGUCGUUUUACAACGUCGUGACUGGGAAAACCCUGGCGUUACCCAACU-3′) in RNA structure buffer (10 mM Tris-HCl [pH 7.0], 0.1M KCl, and 10 mM MgCl_2_) was denatured at 95°C for 2 minutes, and it was chilled on ice for 3 minutes and then at room temperature (RT) for 30 minutes to allow proper secondary structure formation. Cells were lysed in the buffer (10 mM Tris-HCl [pH 7.0], 1.5 mM MgCl_2_, 10 mM KCl, 0.5 mM DTT, and 0.25% NP-40) containing protease inhibitor cocktail and PMSF for 10 minutes on ice. The extract was cleared by centrifugation at 2500*g* at 4°C for 20 minutes. Folded RNA was then mixed with the resulting supernatant in 500 μL RIP buffer (150 mM KCl, 25 mM Tris-HCl [pH 7.4], 0.5 mM DTT, and 0.5% NP-40) and then incubated at RT for 1 hour. Subsequently, 50 μL washed streptavidin beads (Invitrogen, catalog 65601) were added and incubated at RT for 1 hour. After washing 5 times with RIP buffer, the beads were boiled in 1× SDS loading buffer, analyzed by SDS-PAGE, and visualized by silver staining. The pulled-down proteins were identified by mass spectrometry–based quantitative proteomics.

### Animal experiment.

All animal experiments were performed following the guideline and protocols approved by the Animal Care & Welfare Committee of Southeast University, China. C57BL/6 female mice aged 6–8 weeks were purchased from University of Yangzhou (Yangzhou, China). All mice were maintained in the animal house facilities of Southeast University. Mice were anesthetized using inhaled isoflurane and oropharyngeally instilled with mascRNA-expressing plasmid or empty plasmid (20 μg per mouse) complexed with in vivo jetPEI (Polyplus, catalog 201-10G). Forty-eight hours later, the mice were treated with oropharyngeal LPS (1 mg/kg body weight) or poly(I:C) (5 mg/kg body weight) for 6 hours. All mice were sacrificed after LPS or poly(I:C) treatment and subjected to analysis of the severity of lung injury.

### Statistics.

The 2-tailed Student’s *t* test was used for comparison between 2 groups in the GraphPad Prism 6.0, and differences with a *P* value less than 0.05 were considered statistically significant.

### Study approval.

All animal experiments were performed following the guideline and protocols approved by the Animal Care & Welfare Committee of Southeast University, China (no. 2017022705).

Other detailed information about experimental design, including reagents, antibodies, plasmid construction, isolation of nuclear and cytoplasmic RNA, mRNA and protein expression assay, cytoplasmic and nuclear protein extraction, immunofluorescence, RNA immunoprecipitation, dual-luciferase reporter assay, ubiquitination assay, and enzyme-linked immunosorbent assay can be found in Supplemental Methods.

## Author contributions

XM conceived and supervised the study. TS, CW, XW, JW, and YH performed experiments. DW provided technical support. XM, TS, and CW analyzed and interpreted data. XM and TS wrote the manuscript. CW contributed to the preparation of the manuscript. TS and CW share co–first author position; the order of the co–first authors is based on their relative contribution to this study. All authors reviewed and approved the manuscript.

## Supplementary Material

Supplemental data

## Version 1 09/28/2021

In-Press Preview

## Version 2 11/08/2021

Electronic publication

## References

[1] Takeuchi O, Akira S (2010). Pattern recognition receptors and inflammation. Cell.

[2] Jiang X, Chen ZJ (2011). The role of ubiquitylation in immune defence and pathogen evasion. Nat Rev Immunol.

[3] Fitzgerald KA, Kagan JC (2020). Toll-like receptors and the control of immunity. Cell.

[4] Brubaker SW (2015). Innate immune pattern recognition: a cell biological perspective. Annu Rev Immunol.

[5] Lamothe B (2008). The RING domain and first zinc finger of TRAF6 coordinate signaling by interleukin-1, lipopolysaccharide, and RANKL. J Biol Chem.

[6] Shi JH, Sun SC (2018). Tumor necrosis factor receptor-associated factor regulation of nuclear factor κB and mitogen-activated protein kinase pathways. Front Immunol.

[7] Zhao W (2012). E3 ubiquitin ligase tripartite motif 38 negatively regulates TLR-mediated immune responses by proteasomal degradation of TNF receptor-associated factor 6 in macrophages. J Immunol.

[8] Wu C (2017). NLRP11 attenuates Toll-like receptor signalling by targeting TRAF6 for degradation via the ubiquitin ligase RNF19A. Nat Commun.

[9] Jiao S (2015). The kinase MST4 limits inflammatory responses through direct phosphorylation of the adaptor TRAF6. Nat Immunol.

[10] Park Y (2020). Destablilization of TRAF6 by DRAK1 suppresses tumor growth and metastasis in cervical cancer cells. Cancer Res.

[11] Yao K (2018). RSK2 is required for TRAF6 phosphorylation-mediated colon inflammation. Oncogene.

[12] Zhang X (2013). UBE2O negatively regulates TRAF6-mediated NF-κB activation by inhibiting TRAF6 polyubiquitination. Cell Res.

[13] Bellet MM (2020). HOPS/Tmub1 involvement in the NF-kB-mediated inflammatory response through the modulation of TRAF6. Cell Death Dis.

[14] Schneider M (2012). The innate immune sensor NLRC3 attenuates Toll-like receptor signaling via modification of the signaling adaptor TRAF6 and transcription factor NF-κB. Nat Immunol.

[15] Du M (2017). The LPS-inducible lncRNA Mirt2 is a negative regulator of inflammation. Nat Commun.

[16] Xie C (2020). A hMTR4-PDIA3P1-miR-125/124-TRAF6 regulatory axis and its function in NF kappa B signaling and chemoresistance. Hepatology.

[17] Wilusz JE (2008). 3’ end processing of a long nuclear-retained noncoding RNA yields a tRNA-like cytoplasmic RNA. Cell.

[18] Gutschner T (2013). MALAT1 — a paradigm for long noncoding RNA function in cancer. J Mol Med (Berl).

[19] Zhao G (2016). The long noncoding RNA MALAT1 regulates the lipopolysaccharide-induced inflammatory response through its interaction with NF-κB. FEBS Lett.

[20] Gast M (2019). Immune system-mediated atherosclerosis caused by deficiency of long non-coding RNA MALAT1 in ApoE-/-mice. Cardiovasc Res.

[21] Gast M (2016). Long noncoding RNA MALAT1-derived mascRNA is involved in cardiovascular innate immunity. J Mol Cell Biol.

[22] Do-Umehara HC (2013). Suppression of inflammation and acute lung injury by Miz1 via repression of C/EBP-δ. Nat Immunol.

[23] Tseng PH (2010). Different modes of ubiquitination of the adaptor TRAF3 selectively activate the expression of type I interferons and proinflammatory cytokines. Nat Immunol.

[24] Lu X (2020). The tRNA-like small noncoding RNA mascRNA promotes global protein translation. EMBO Rep.

[25] Magee R, Rigoutsos I (2020). On the expanding roles of tRNA fragments in modulating cell behavior. Nucleic Acids Res.

[26] Kumar P (2016). Biogenesis and function of transfer RNA-related fragments (tRFs). Trends Biochem Sci.

[27] Gohda J (2004). Cutting edge: TNFR-associated factor (TRAF) 6 is essential for MyD88-dependent pathway but not toll/IL-1 receptor domain-containing adaptor-inducing IFN-beta (TRIF)-dependent pathway in TLR signaling. J Immunol.

[28] Häcker H (2006). Specificity in Toll-like receptor signalling through distinct effector functions of TRAF3 and TRAF6. Nature.

[29] Jiang Z (2004). Toll-like receptor 3-mediated activation of NF-kappaB and IRF3 diverges at Toll-IL-1 receptor domain-containing adapter inducing IFN-beta. Proc Natl Acad Sci U S A.

[30] Doyle SE (2003). Toll-like receptor 3 mediates a more potent antiviral response than Toll-like receptor 4. J Immunol.

[31] Jakimovski D (2018). Interferon β for multiple sclerosis. Cold Spring Harb Perspect Med.

[32] Kaur A, Goggolidou P (2020). Ulcerative colitis: understanding its cellular pathology could provide insights into novel therapies. J Inflamm (Lond).

[33] Mannon PJ (2011). Suppression of inflammation in ulcerative colitis by interferon-β-1a is accompanied by inhibition of IL-13 production. Gut.

[34] Wang Y (2013). The autoimmunity-associated gene PTPN22 potentiates toll-like receptor-driven, type 1 interferon-dependent immunity. Immunity.

[35] Borden EC (2019). Interferons α and β in cancer: therapeutic opportunities from new insights. Nat Rev Drug Discov.

[36] Liu W (2020). LncRNA Malat1 inhibition of TDP43 cleavage suppresses IRF3-initiated antiviral innate immunity. Proc Natl Acad Sci U S A.

[37] Chen R (2017). Quantitative proteomics reveals that long non-coding RNA MALAT1 interacts with DBC1 to regulate p53 acetylation. Nucleic Acids Res.

[38] Han SP (2010). Functional diversity of the hnRNPs: past, present and perspectives. Biochem J.

[39] Xiao X (2009). Splice site strength-dependent activity and genetic buffering by poly-G runs. Nat Struct Mol Biol.

[40] Bouchareychas L (2017). Critical role of LTB4/BLT1 in IL-23-induced synovial inflammation and osteoclastogenesis via NF-κB. J Immunol.

