# Supplementary material for: The small RNA mascRNA differentially regulates TLR-induced proinflammatory and antiviral responses
Source: JCI Insight. 2021 Nov 8;6(21):e150833. doi: 10.1172/jci.insight.150833 (PMC8663567; doi:10.1172/jci.insight.150833)
Supplement: Supplemental data [file jciinsight-6-150833-s216.pdf]

Supplemental Information for

The small RNA mascRNA differentially regulates TLR-induced proinflammatory and antiviral responses

Tao Sun[1], Chunxue Wei[1], Daoyong Wang[2], Xuxu Wang[2], Jiao Wang[1], Yuqing Hu[2] and Xiaohua Mao[1,2]

[1]School of Life Science and Technology, Key Laboratory of Ministry of Education for Developmental Genes and Human Disease, Southeast University, Nanjing, Jiangsu, China

[2]Department of Biochemistry and Molecular Biology, School of Medicine, Southeast University, Nanjing, Jiangsu 210009, China

Authorship note: TS and CW contributed equally to this work.

Address correspondence to: Xiaohua Mao, Department of Biochemistry and Molecular Biology, School of Medicine, Southeast University, Nanjing, Jiangsu 210009, China. Phone: (25) 83272474; Email: xmao@seu.edu.cn.

Methods

Reagents and antibodies

E. coli LPS (3023), phorbol-12-myristate-13-acetate (PMA) (P8139), thioglycollate medium (T9032) and 5Z-7-Oxozeaenol (O9890) were obtained from Sigma Aldrich. NH_4Cl (ST2030), Cycloheximide (SC0353) and G-418 (ST081) were obtained from Beyotime Biotechnology (Shanghai, China). Other reagents used in this study include Pam3CSK4 (InvivoGen, Cat# tlrl-pms), poly(I:C) (InvivoGen, Cat# tlrl-pic-5), 3-Methyladenine (Selleck, Cat# S2767).

Primary antibodies used include: anti-TNF (ab183218), anti-IL-6 (ab214429), anti-IKK (ab178870) and anti-Ub-k48 (ab140601), anti-Ub-K63 (ab79434) and anti-IRF3 (ab68481) (Abcam); anti-Lamin B (12987-1-AP), anti-hnRNP H1 (14774-1-AP), anti-hnRNP F (14974-1-AP), anti-β-actin (20536-1-AP), anti-TRAF6 (66498-1-lg) , anti-Ubiquitin (10201-2-AP) (Proteintech) ; anti-TRIF (sc-514384), anti-TAK1 (sc-7967), anti-TLR4 (sc-293072), anti-MyD88 (sc-74532), anti-IRAK1 (sc-5288), anti-NF-κB p65 (sc-514451), anti-TRAF3 (sc-6933) and anti-TRIF (sc-514384) (Santa Cruz Biotechnology); anti-Phospho-IκBα (9246), anti-Phospho-p38 MAPK (4511), anti-p38 MAPK (8690), anti-Phospho-JNK (4668), anti-JNK (9252), anti-Phospho-ERK (4370), anti-ERK (4695), anti-Phospho-IKK (2697), anti-IκBα (4814), anti-IL-6 (12912), anti-Phospho-IRF3 (4947), anti-Phospho-STAT1 (9177) and anti-STAT1 (9172) (Cell Signaling Technology). HRP-conjugated Affinipure Goat Anti-Rabbit IgG (SA00001-2) and HRP-conjugated Affinipure Goat Anti-Mouse IgG (SA00001-1) (Proteintech) secondary antibodies were used.

Plasmid constructions

Using the genomic DNA extracted from RAW264.7 cells as a template, the mouse *Tnf* promoter from -1153 to +66 and *Il6* promoter from -731 to +68 were amplified by PCR. *Tnf* or *Il6* promoter luciferase reporter plasmid was constructed by inserting *Tnf* or *Il6* promoter sequence into the *Kpn* I and *Hin*d III sites (upstream of the luciferase sequence) of pGL-4.17 (Promega). The primers used in plasmid construction are listed in Supplemental Table 1. mascRNA-expressing vector (pGV-mascRNA) was constructed by inserting mouse mascRNA full-length sequence into the *Bam*H I and *Hin*d III sites (downstream of U6 promoter) of GV251 vector (purchased from GeneChem Co. Ltd (Shanghai, China)). All constructs were verified by DNA sequencing.

Isolation of nuclear and cytoplasmic RNA

Isolation of nuclear and cytoplasmic RNA was performed as described (1). Briefly, RAW264.7 cells were seeded into 60 mm dishes at 5×10^6 cells per dish. After 24 h, cells were washed with cold PBS and lysed with 1 mL PBS containing 0.1% NP-40. After reserving 500 μL lysate as total fraction, the remaining 500 μL lysate was centrifuged at 3000 rpm for 5 min at 4 ℃, and the supernatant was collected as cytoplasmic fraction. After washing the precipitate twice with PBS containing 0.1% NP-40, the pellet was lysed with 500 μL PBS containing 0.5% NP-40 and considered as nuclear fraction. RNA from all parts of cells was extracted using the TRIzol Reagent (Takara, Cat# D9108) according to the manufacturers' protocol.

RT-qPCR

Total RNA was extracted from cells or tissues using the TRIzol Reagent according to the manufacturers' protocol. Total RNA was reverse-transcribed with mascRNA and U6 specific primers using PrimeScript RT reagent Kit, followed by real-time quantitative PCR

(RT-qPCR) using TaqMan probe qPCR kit (Takara, Cat# RR390A). For the expression analysis of other genes, total RNA was reversely transcribed into first-strand cDNA using PrimeScript RT reagent Kit with gDNA Eraser (Takara, Cat# RR047A), followed by qPCR using TB Green qPCR kit (Takara, Cat# RR820A). Gene expression was normalized to that of β-actin or GAPDH. The primers and Taqman probes for real-time PCR are shown in Supplemental Table 1.

Cytoplasmic and nuclear protein extraction

Cells were washed with cold PBS and lysed with cytoplasmic lysate buffer (20 mM Tris-HCl [pH 7.5], 10 mM NaCl, 3 mM $MgCl_2$ and 0.5% NP-40) containing protease inhibitor cocktail (Roche, Cat# 05892791001) and PMSF (Beyotime Biotechnology, China, Cat# ST505) for 15 min on ice and then centrifuged for 10 min at 3000 rpm, 4 ℃. The supernatant was collected as cytoplasmic fraction and the remainder was considered as a nuclear pellet. The nuclear pellet was washed with cold PBS and lysed with nuclear lysate buffer (25 mM Tris-HCl [pH 7.5], 150 mM NaCl, 1% NP-40, 1% sodium deoxycholate and 0.1% SDS) containing protease inhibitor cocktail and PMSF for 30 min on ice and then centrifuged for 20 min at 13000 rpm, 4 ℃. The supernatant was collected as a nuclear fraction.

Immunofluorescence

THP-1 cells grown on glass coverslips were fixed in 4% paraformaldehyde for 15 min and permeabilized with 0.1% Triton X-100 for 40 min. Then cells were washed three times with PBS and blocked with 5% (wt/vol) BSA in PBS for 10 min. The cells were washed three times with PBS and incubated with anti-NF-κB p65 (Santa Cruz Biotechnology, Cat# sc-514451) overnight at 4 ℃. Then cells were washed three times with PBS and incubated

with fluorescent-labeled secondary antibody (Proteintech, SA00013-3) for 1 h at room temperature (RT). After three times washing with PBS, DAPI was used to stain nuclei for 30 min in the dark at RT. Staining was visualized under a confocal laser microscope.

Western blot

Cells were washed with cold PBS and lysed with lysis buffer (10 mM Tris-HCl [pH 8.0], 1mM EDTA, 0.5mM EGTA, 1% Triton X-100, 150 mM NaCl, 0.1% SDS, 0.2% deoxycholate acid, 50mM NaF and 1mM Na_3VO_4) containing protease inhibitor cocktail and PMSF. For western blotting, twenty microgram proteins per sample were separated by SDS-polyacrylamide gel electrophoresis (PAGE) and transferred to nitrocellulose membranes (Millipore, Cat# HATF00010). Each membrane was blocked with 5% (wt/vol) non-fat dry milk in TBS-T buffer (20 mM Tris-HCl [pH 7.4], 150 mM NaCl and 0.05 % Tween-20). After three washes in TBS-T buffer, the membrane was incubated with indicated primary antibodies in TBS-T containing 5% BSA at 4 ℃ overnight with gentle shaking. Then, after three washes in TBS-T, membranes were immunoreacted with the secondary antibodies in TBS-T containing 3% (wt/vol) non-fat dry milk at RT for 1 h. After three washes in TBS-T, the specific bands were detected with a Tanon-5200 gel imaging system (Shanghai, China). Images have been cropped for presentation.

RNA immunoprecipitation (RIP)

RIP was done as described previously with minor modifications (2). Briefly, Cells were washed with cold PBS and lysed with RIPA buffer (40 mM Tris, 120 mM NaCl, 1% Triton X-100, 1 mM NaF, and 1 mM Na_3VO_4) containing protease inhibitor cocktail and PMSF. After incubation at 4 ℃ for 20 min, the lysates were centrifuged at 13200 rpm at 4 ℃ for 20 min. The supernatants were incubated overnight with anti-hnRNP F/H antibody (Santa

Cruz Biotechnology, Cat# sc-32310) or negative control IgG antibody (Millipore, Cat# 17700) followed by 2 h incubation with 20 μL Protein A/G PLUS-Agarose (Santa Cruz Biotechnology, Cat# sc-2003) at 4 ℃. The beads were spun down at 1000 × *g* and washed with RIPA buffer five times. The co-precipitated RNAs were extracted with the TRIzol reagent, and the abundance of mascRNA was analyzed by Taqman probe RT-qPCR. U6 was served as irrelevant RNA controls.

Dual-luciferase reporter assay

To analyze the effect of mascRNA on *Tnf* and *Il6* promoter activities, MEFs were seeded into 24-well plates at 3×10^4 cells per well. After 24 h, they were transfected with mascRNA ASO or NC ASO. 12 h later, cells were again cotransfected with a reporter plasmid containing either *Tnf* or *Il6* promoter-driven luciferase reporter and a Renilla luciferase reporter plasmid. At 48 h after transfection, cells were stimulated with or without LPS for 6 h. To analyze the effect of hnRNP H or F on *Tnf* and *Il6* promoter activities, MEFs were seeded into 24-well plates at 5×10^4 cells per well. After 24 h, MEFs were cotransfected with a reporter plasmid containing a *Tnf* or *Il6* promoter-driven luciferase reporter, a Renilla luciferase reporter plasmid, and an indicated siRNA (H siRNA or F siRNA). At 48 h after transfection, cells were stimulated with or without LPS for 6 h. Luciferase activity was measured with Dual-Luciferase Reporter Assay System (Vazyme, China, Cat# DL101-01) according to the manufacturers' instruction. Samples were analyzed in triplicate and normalized to Renilla luciferase activity.

Ubiquitination assay

Cells were lysed with SDS lysis buffer (50 mM Tris-HCl [pH 6.8], 150 mM NaCl, 10% glycerol and 1% SDS) containing protease inhibitor cocktail tablets and PMSF, and

denatured by boiling for 5 min. The boiled lysates were diluted with dilution buffer (10 mM Tris-HCl [pH 8.0], 150 mM NaCl, 2 mM EDTA and 1% Triton X-100). After incubation at 4 ℃ for 20 min, the lysates were centrifuged at 13200 rpm at 4 ℃ for 20 min. The supernatants incubated 2 h with 1-2 μg indicated antibody followed by 2 h incubation with 20 μL Protein A/G PLUS-Agarose at 4 ℃. After washing five times with dilution buffer, immunoprecipitated proteins were boiled with 2 × SDS loading buffer, and analyzed by western blot with antibodies specific for ubiquitin. Antibodies used for immunoprecipitation include anti-TRAF6 antibody (Santa Cruz Biotechnology, sc-8409) and anti-TRAF3 antibody (Santa Cruz Biotechnology, sc-6933).

Enzyme-linked immunosorbent assay

The concentration of TNF, IL-6, IL-1β (Biolegend) and IFN-β (Cusabio, Wuhan, China) in cell culture supernatants or BALFs was determined using ELISA assay kits according to the manufacturers' instruction.

Measuring activity of MPO

The myeloperoxidase (MPO) activity in mice bronchoalveolar lavage fluids (BALFs) was measured using MPO assay kit (Nanjing Jiancheng Bioengineering Institute, China, Cat# A044-1-1) according to the manufacturers' instruction.

Supplemental references

1. W. Liu, et al. LncRNA Malat1 inhibition of TDP43 cleavage suppresses IRF3-initiated antiviral innate immunity. *Proc Natl Acad Sci U S A.* 2020;117, 23695-23706.

2. R. Chen, et al. Quantitative proteomics reveals that long non-coding RNA

MALAT1 interacts with DBC1 to regulate p53 acetylation. *Nucleic Acids Res.* 2017;45, 9947-9959.

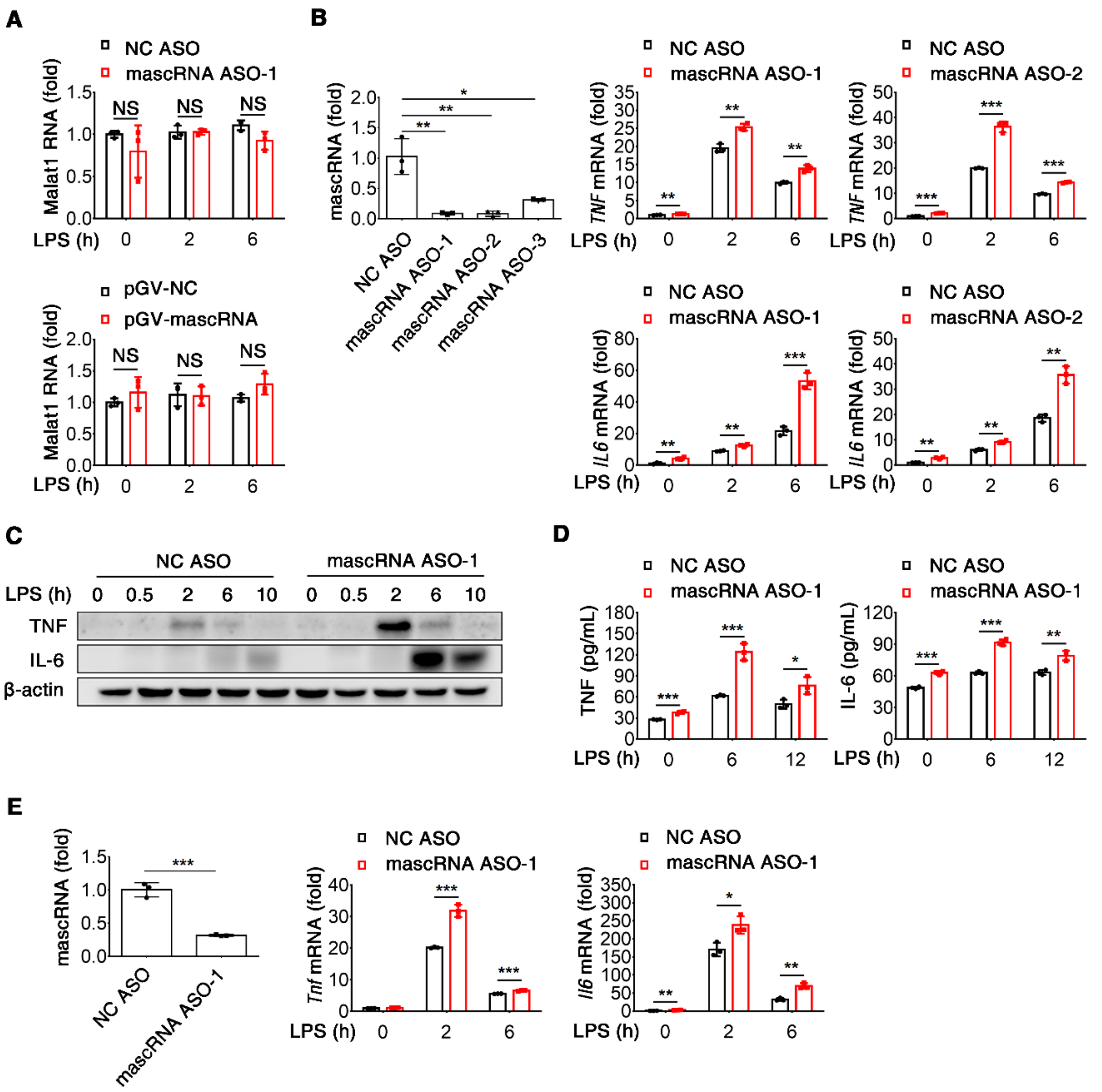

Supplemental Figure 1 (related to Figure 1)

(**A**) mascRNA does not alter Malat1 abundance. mascRNA-knockdown (top) or mascRNA-overexpressing (bottom) RAW264.7 cells were stimulated with LPS for the indicated times, followed by qPCR analysis of Malat1 abundance.

(**B-D**) mascRNA knockdown increases TNF and IL-6 expression in THP-1 macrophages in response to LPS challenge. The knockdown efficiency of 3 antisense oligonucleotides (ASOs) and the effect of two ASOs (ASO-1 and -2) on *TNF* and *IL6* mRNA expression upon LPS stimulation were measured by qPCR (**B**), cellular TNF and IL-6 precursors analyzed by immunoblot (**C**), and secreted TNF and IL-6 measured by ELISA (**D**).

(**E**) mascRNA knockdown increases *Tnf* and *Il6* mRNA expression in mouse peritoneal macrophages (MPMs) in response to LPS challenge. The knockdown efficiency of mascRNA ASO-1 and the mRNA

abundance of *Tnf* and *Il6* were measured by qPCR. mascRNA ASO-1, -2 and -3 represent 3 ASOs targeting mouse or human mascRNA, respectively.

pGV-mascRNA, the vector overexpressing mascRNA; NC, negative control. Data shown in **A**, **B** and **E** are the mean ± SD of triplicate wells. * *P* <0.05; ** *P* <0.01; *** *P* <0.001 (2-tailed Student's *t* test). Data shown in **A**, **B**, **C** and **E** are representatives of two independent experiments.

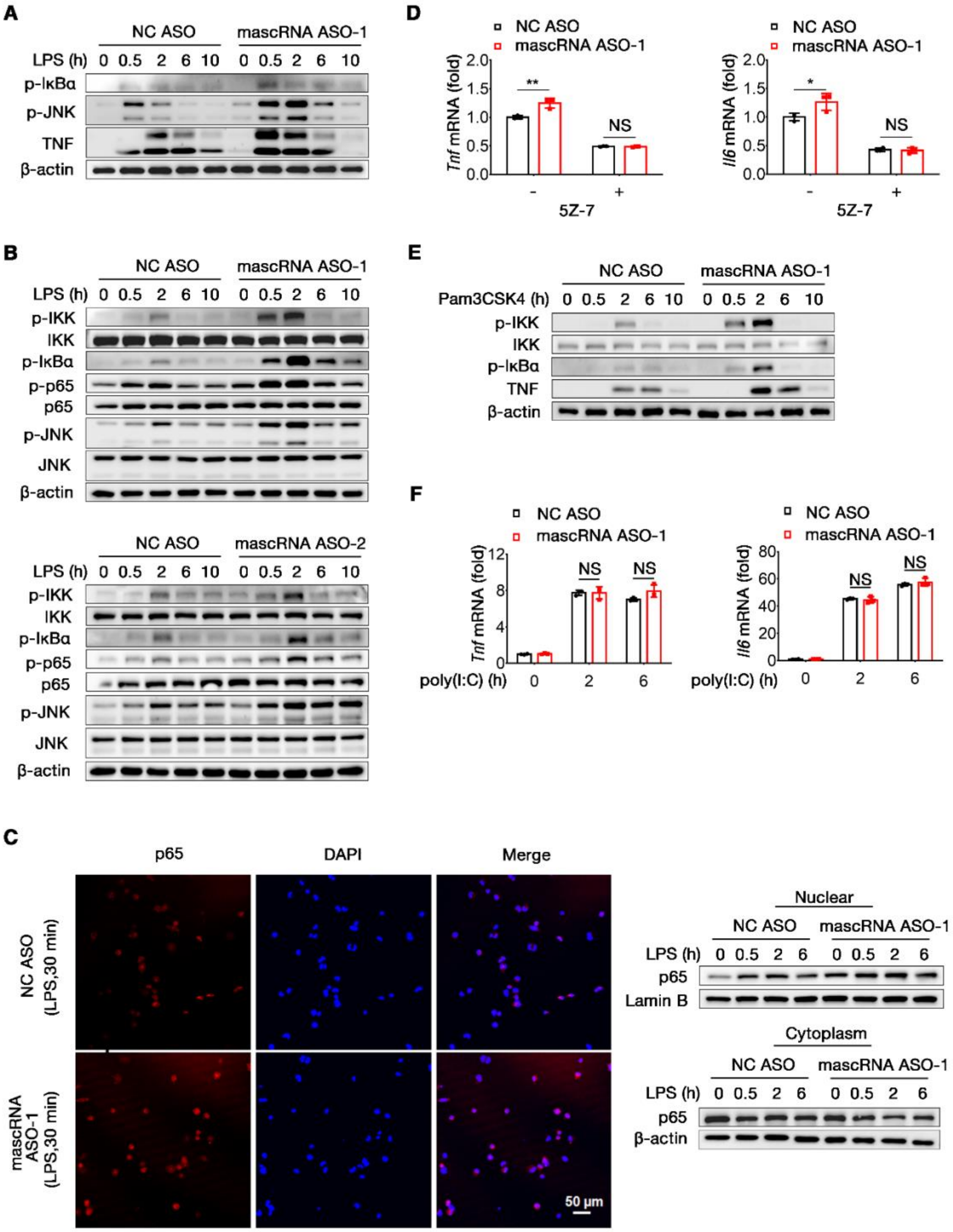

Supplemental Figure 2 (related to Figure 2)

(**A**) Immunoblot analysis of p-IκBα, p-JNK and cellular TNF precursor in mascRNA-knockdown mouse peritoneal macrophages stimulated with LPS for the indicated times.

(**B**) Effects of mascRNA knockdown on NF-κB and MAPK signaling in LPS-stimulated THP-1 macrophages. mascRNA was knocked down by two ASOs (ASO-1 and -2). Key molecules in the NF-κB and MAPK signaling pathways were analyzed by immunoblot.

(**C**) p65 nuclear translocation in THP-1 macrophages assayed by immunofluorescent confocal microscopy (left panel) and nuclear/cytoplasmic fractionation followed by immunoblot (right panel).

(**D**) mascRNA inhibits TAK1 activity to suppress proinflammatory cytokine expression. mascRNA-knockdown MPMs were pretreated with or without 5Z-7 for 30 min prior to 2-h (*Tnf*) or 6-h (*Il6*) LPS challenge, followed by qPCR analysis of *Tnf* and *Il6* expression.

(**E**) Immunoblot analysis of p-IKK, total IKK, p-IκBα and cellular TNF precursor in mascRNA-knockdown THP-1 macrophages treated with Pam3CSK4.

(**F**) qPCR analysis of *Tnf* and *Il6* expression in mascRNA-knockdown RAW264.7 macrophages treated with poly(I:C).

NC, negative control. Data shown in **D** and **F** are the mean ± SD of triplicate wells. **P* <0.05; ***P* <0.01; NS, not significant (2-tailed Student's *t* test). Data shown in **A**, **B** and **E** are representatives of two independent experiments.

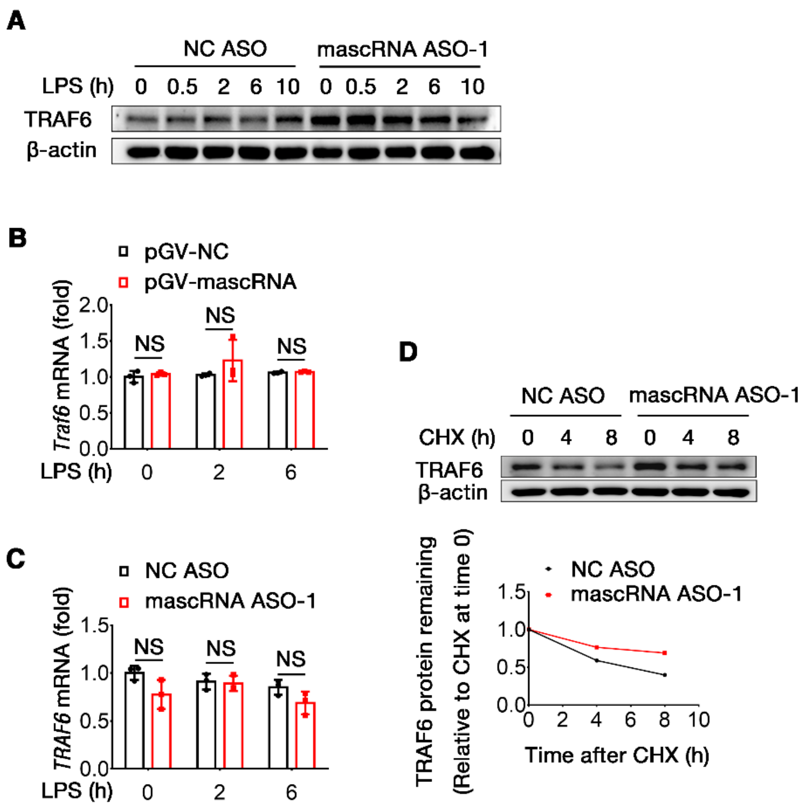

Supplemental Figure 3 (related to Figure 3)

(**A**) mascRNA knockdown increases TRAF6 protein abundance. mascRNA-knockdown THP-1 macrophages were stimulated with LPS for the indicated times, followed by immunoblot analysis of TRAF6 protein.

(**B**) mascRNA overexpression does not change *Traf6* mRNA level. mascRNA-overexpressing RAW264.7 macrophages were stimulated with LPS for the indicated times, followed by qPCR analysis of *Traf6* mRNA abundance.

(**C**) mascRNA knockdown does not change *TRAF6* mRNA level. mascRNA-knockdown THP-1 macrophages were stimulated with LPS for the indicated times, followed by qPCR analysis of *TRAF6* mRNA abundance.

(**D**) mascRNA promotes degradation of TRAF6 protein. mascRNA-knockdown THP-1 macrophages were treated with 40 ng/mL cycloheximide (CHX) for the indicated times, followed by immunoblot analysis.

NC, negative control. Data shown in **B** and **C** are the mean ± SD of triplicate wells. NS, not significant (2-tailed Student's *t* test). Data shown in **A** and **D** are representatives of two independent experiments.

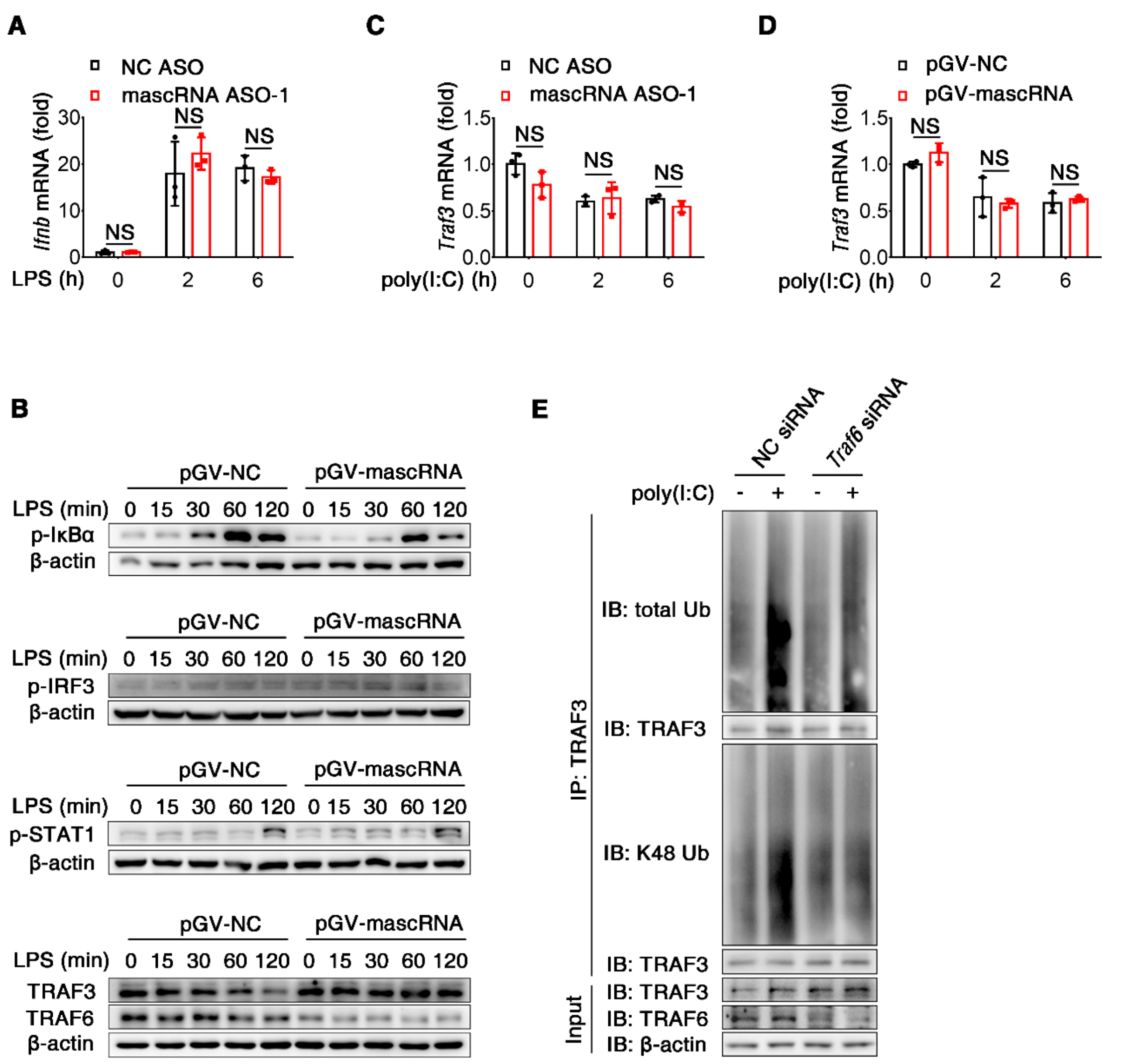

Supplemental Figure 4 (related to Figure 5)

(**A**) qPCR analysis of *Ifnb* expression in mascRNA-knockdown RAW264.7 cells stimulated with LPS for the indicated times.

(**B**) mascRNA-overexpressing RAW264.7 cells were stimulated with LPS for the indicated times, followed by immunoblot analysis of indicated proteins.

(**C** and **D**) mascRNA-knockdown (**C**) or mascRNA-overexpressing (**D**) RAW264.7 cells were stimulated with poly(I:C) for the indicated times, followed by qPCR analysis of *Traf3* expression.

(**E**) TRAF6 promotes poly(I:C)-induced K48-linked ubiquitination of TRAF3. RAW264.7 cells transfected with TRAF6 siRNA or NC siRNA were stimulated with poly(I:C) for 15 min, immunoprecipitated with an anti-TRAF3 antibody, and analyzed by immunoblot using indicated antibodies. Bottom, immunoblot analysis of TRAF3, TRAF6 and β-actin in lysates without immunoprecipitation.

NC, negative control. Data shown in **A**, **C** and **D** are the mean ± SD of triplicate wells. NS, not significant (2-tailed Student's *t* test). Data shown in **E** are representative of 2 independent experiments.

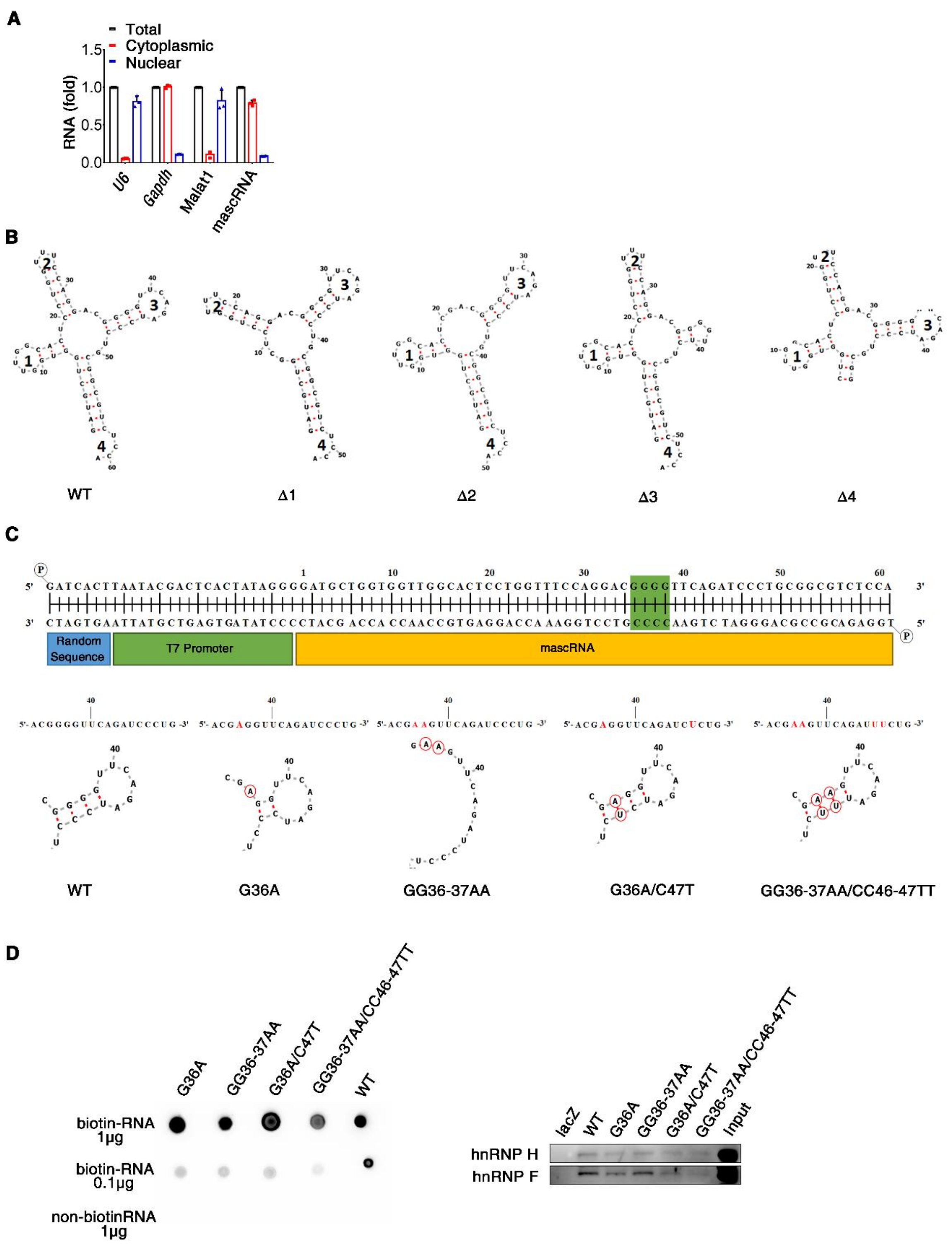

Supplemental Figure 5 (related to Figure 7)

(**A**) qPCR analysis of relative levels of U6, *Gapdh*, Malat1 and mascRNA in subcellular fractions of RAW264.7 cells.

(**B**) Predicted secondary structures of human wildtype mascRNA and four secondary structure-based mascRNA deletion mutants (Δ1-4).

(**C**) Top: The DNA template used to construct human mascRNA mutants. The G-rich motif is shaded green. The first nucleotide of mascRNA is designated 1. Bottom: The location of mutated nucleotides and predicted secondary structures of 4 point mutants of mascRNA. Mutated nucleotides are shown in red or circled. G36A and GG36-37AA are two point mutations in the G-rich motif which are predicted to alter local stem-loop structures. G36A/C47T and GG36-37AA/CC46-47TT are compensatory mutations for G36A and GG36-37AA, respectively.

(**D**) The G-rich motif in mascRNA is not responsible for interacting with hnRNP H1 and F. Left: Biotin labeling efficiency of wildtype mascRNA and its four point mutants determined by dot blot. Right: Immunoblot of hnRNP H and F in the samples pulled down by biotinylated wildtype mascRNA or its four point mutants from cytoplasmic lysates of THP-1 macrophages. A 61-nt *lacZ* mRNA fragment was used as a negative control.

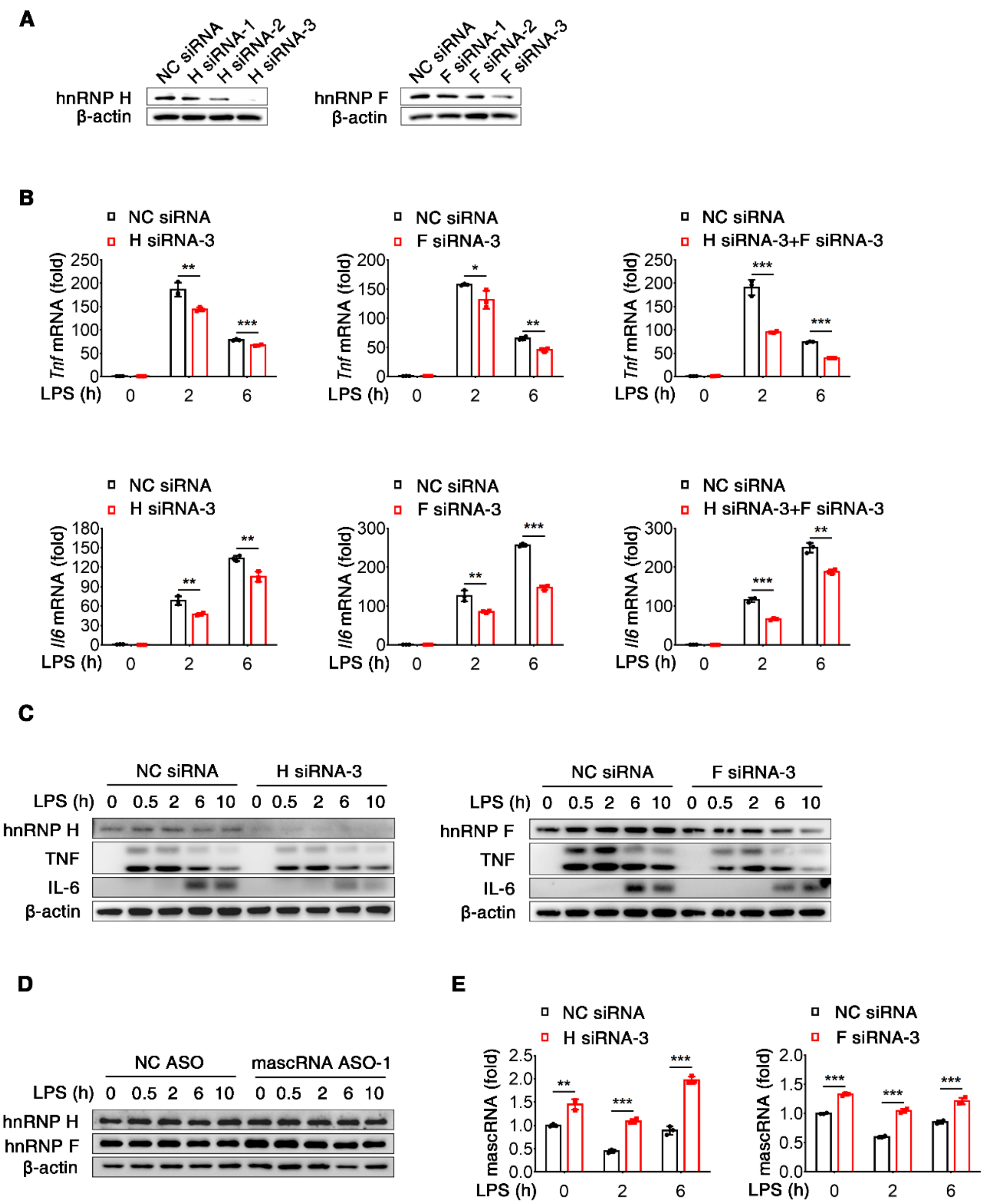

Supplemental Figure 6 (related to Figure 8)

(**A**) Knockdown efficiency of siRNA targeting *hnRNP H* or *hnRNP F*. RAW264.7 cells were transfected with three siRNAs against mouse *hnRNP H* or *hnRNP F*, immunoblot analysis was performed 36 h after transfection.

(**B** and **C**) Knockdown of hnRNP H or F decreases TNF and IL-6 mRNA and protein abundance in mouse peritoneal macrophages (MPMs). MPMs were transfected with *hnRNP H*, *hnRNP F*, or mixed

hnRNP H/F siRNA. 36 h after transfection, cells were stimulated with LPS for the indicated times, followed by qPCR analysis of *Tnf* and *Il6* mRNA (**B**) as well as immunoblot analysis of cellular TNF and IL-6 proteins (**C**).

(**D**) Knockdown of mascRNA does not affect hnRNP H and F protein levels. RAW264.7 cells were transfected with a mascRNA ASO and stimulated with LPS for the indicated times, followed by immunoblot analysis of hnRNP H or hnRNP F protein abundance.

(**E**) Knockdown of hnRNP H or F increases mascRNA abundance. MPMs were transfected with *hnRNP H* or *hnRNP F* siRNA and stimulated with LPS for the indicated times, followed by qPCR analysis of mascRNA abundance.

NC, negative control. Data shown in **B** and **E** are the mean ± SD of triplicate wells. * *P* <0.05; ** *P* <0.01; *** *P* <0.001 (2-tailed Student's *t* test). Data shown in **A**, **C, D** and **E** are representatives of two independent experiments.

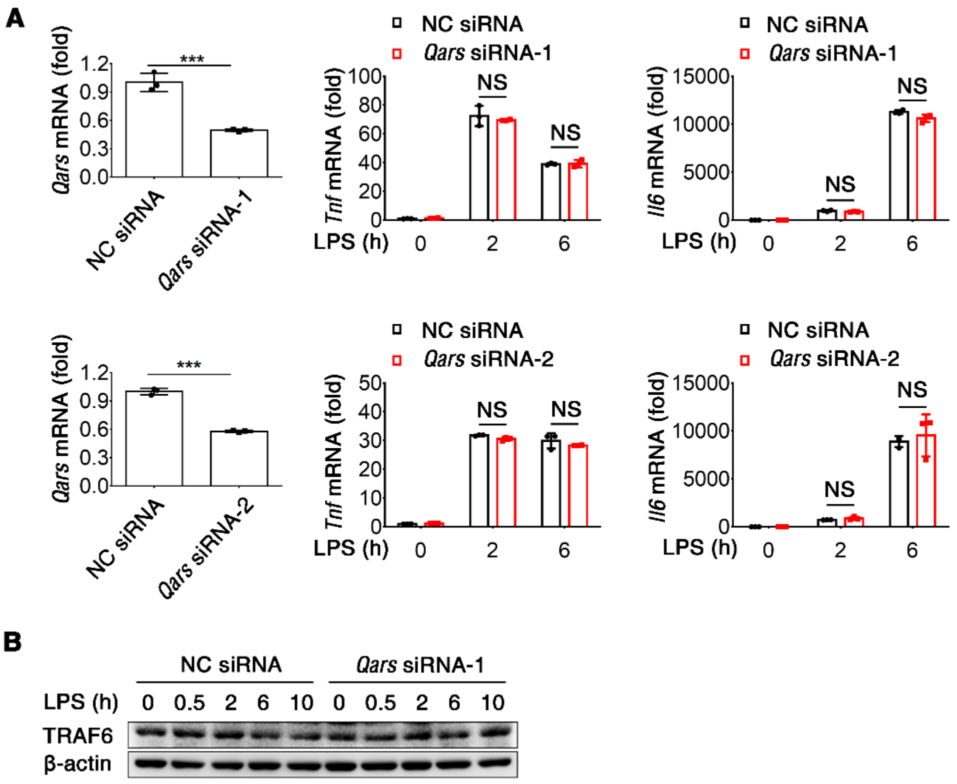

Supplemental Figure 7

Knockdown of glutaminyl-tRNA synthetase (QARS) does not alter proinflammatory cytokine expression and TRAF6 protein abundance in RAW264.7 cells in response to LPS challenge.

(**A**) The knockdown efficiency of two siRNAs (siRNA-1 and -2) targeting *Qars* and the mRNA abundance of *Tnf* and *Il6* measured by qPCR. Data are the mean ± SD of triplicate wells. ****P* <0.001; NS, not significant (2-tailed Student's *t* test).

(**B**) Immunoblot analysis of TRAF6 protein in QARS-knockdown RAW264.7 cells stimulated with LPS for the indicated times.

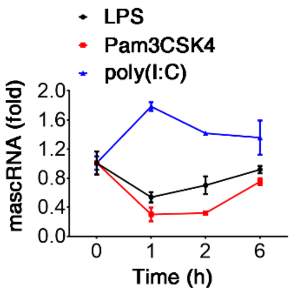

Supplemental Figure 8

The TLR2 ligand Pam3CSK4 induces further downregulation of mascRNA compared to the TLR4 ligand LPS. RAW264.7 cells were stimulated with Pam3CSK4, LPS, or poly(I:C) for the indicated times, followed by qPCR analysis of mascRNA abundance. Data are mean ± SD of triplicate wells and are representative of two independent experiments.

Supplemental Table 1**. Oligonucleotides (5′ to 3′)***

**Primers for constructing luciferase reporter plasmids**	
Mouse *Tnf* (F)	CTGGTACCTGTCTGCTTGTGTCTGT
Mouse *Tnf* (R)	CGCAAGCTTGTTCTGGAGTTTCTGTTCT
Mouse *Il6* (F)	CTGGTACCGAATAGGCTTGGACTTGGAA
Mouse *Il6* (R)	CGCAAGCTTGGAATTGACTATCGTTCTTGG
**Phosphorothioate ASO† and siRNA**	
Human mascRNA ASO-1	mCmCmAmGmGdAdGdTdGdCdCdAdAdCdCmAmCmCmAmG
Human mascRNA ASO-2	mAmAmCmCmCdCdGdTdCdCdTdGdGdAdAmAmCmCmAmG
Human mascRNA ASO-3	mUmGmGmAmAdAdCdCdAdGdGdAdGdTdGmCmCmAmAmC
Mouse mascRNA ASO-1	mAmAmCmCmCdCdGdTdCdCdTdGdGdAdAmAmCmCmAmG
Mouse mascRNA ASO-2	mCmCmAmGmGdAdGdTdGdCdCdAdGdCdCmAmCmCmAmG
Mouse mascRNA ASO-3	mGmAmCmAmCdCdGdCdAdGdGdGdAdCdCmTmGmAmAmC
NC ASO	mGmCmGmUmAdTdTdAdTdAdGdCdCdGdAmUmUmAmAmC
Mouse *hnRNP H1* siRNA-1	GCUGAAGUUAGAACUCAUU
Mouse *hnRNP H1* siRNA-2	CCGGACACUGUGUACACAU
Mouse *hnRNP H1* siRNA-3	GCAGCUGAGUGGUGGUUAU
Mouse *hnRNP F* siRNA-1	CCUCUCCGACUGCACAAUU
Mouse *hnRNP F* siRNA-2	CCGGAGGUACAUUGGCAUU
Mouse *hnRNP F* siRNA-3	GCCAACAUGCAGCACAGAU
Mouse *Traf6* siRNA	CGUCCUUUCCAGAAGUGCC
Mouse *Qars* siRNA-1	GUGGAGUCCACCAUAAAUA
Mouse *Qars* siRNA-2	GAGCGCUGCUCUUGAAUAU
NC siRNA	UUCUCCGAACGUGUCACGU
**qPCR primers**	
Human *TNF* (F)	TCCTTCAGACACCCTCAACC
Human *TNF* (R)	AGGCCCCAGTTTGAATTCTT
Human *IL6* (F)	TACCCCCAGGAGAAGATTCC
Human *IL6* (R)	TTTTCTGCCAGTGCCTCTTT
Human *TRAF6* (F)	GGGGACAATCCATAAGAGCA
Human *TRAF6* (R)	CTGCAAAGCCTGCATCATAA
Human *β-actin* (F)	GTCATTCCAAATATGAGATGCGT
Human *β-actin* (R)	GCTATCACCTCCCCTGTGTG
Human mascRNA (F)	GATGCTGGTGGTTGGCACTC
Human mascRNA (R)	TGGAGACGCCGCAGGGAT
Mouse *Tnf* (F)	TGCCTATGTCTCAGCCTCTTC
Mouse *Tnf* (R)	GAGGCCATTTGGGAACTTCT
Mouse *Il6* (F)	GAGGATACCACTCCCAACAGACC
Mouse *Il6* (R)	AAGTGCATCATCGTTGTTCATACA
Mouse *Traf6* (F)	CTCAGCGCTGTGCAAACTATATATCCC
Mouse *Traf6* (R)	GGCGTATTGTACCCTGGAAGGG
Mouse *Ifnb* (F)	GTACGTCTCCTGGATGAACTCC
Mouse *Ifnb* (R)	CCACGTCAATCTTTCCTCTTGC
Mouse *Cxcl10* (F)	GTGTGTGCGTGGCTTCACT
Mouse *Cxcl10* (R)	GAGATCATTGCCACGATGAA
Mouse *Ccl5* (F)	GCTGCTTTGCCTACCTCTCC
Mouse *Ccl5* (R)	TCGAGTGACAAACACGACTGC
Mouse *Ifit3* (F)	CCTACATAAAGCACCTAGATGGC
Mouse *Ifit3* (R)	ATGTGATAGTAGATCCAGGCGT
Mouse *Traf3* (F)	GCAGCGTTCAGACTCTTC
Mouse *Traf3* (R)	GTTGCTCCACTCCTTCAG
Mouse *Gapdh* (F)	TGTGTCCGTCGTGGATCTGA
Mouse *Gapdh* (R)	TTGCTGTTGAAGTCGCAGGAG

Mouse mascRNA (F)	GACGCTGGTGGCTGGCACT
Mouse mascRNA (R)	TGGAGACACCGCAGGGAC
Mouse Malat1 (F)	CATGGCGGAATTGCTGGTA
Mouse Malat1 (R)	TGCCAACAGCATAGCAGTA
Mouse *Qars* (F)	CACCAGAGGCTATCAACAA
Mouse *Qars* (R)	GCATCATTCAGCACATCAC
U6 (F)	CTCGCTTCGGCAGCACA
U6 (R)	AACGCTTCACGAATTTGCGT
Human mascRNA TaqMan Probe	CTGGTTTCCAGGACGGGGTTCAGATCCCT
Mouse mascRNA TaqMan Probe	CTGGTTTCCAGGACGGGGTTCAGGTCC
U6 Taqman Probe	CCATGCTAATCTTCTCTGTCTCGTTCCA

In Vitro transcription template and primers

mascRNA Δ1 (S)	GATCACTTAATACGACTCACTATAGGGGATGCTGGCTCCTGGTTC
mascRNA Δ1 (A)	TGGAGACGCCGCAGGGATCTGAACCCCGTCCTGGAAACCAGGAGCCAGCA
mascRNA Δ2 (S)	GATCACTTAATACGACTCACTATAGGGGATGCTGGTGGTTGGCACTCGAC
mascRNA Δ2 (A)	TGGAGACGCCGCAGGGATCTGAACCCCGTCGAGTGCCAACCACCAGCATC
mascRNA Δ3 (S)	GATCACTTAATACGACTCACTATAGGGGATGCTGGTGGTTGGCACTCCTG
mascRNA Δ3 (A)	TGGAGACGCCGCAGAACCCCGTCCTGGAAACCAGGAGTGCCAACCACCAGCATC
mascRNA Δ4 (S)	GATCACTTAATACGACTCACTATAGGGGCTGGTGGTTGGCACTCCTG
mascRNA Δ4 (A)	GCCGCAGGGATCTGAACCCCGTCCTGGAAACCAGGAGTGCCAACCACCAG
mascRNA G36A (S)	GATCACTTAATACGACTCACTATAGGGGATGCTGGTGGTTGGCACTCCTGGTTTCCAGGACGAG
mascRNA G36A (A)	TGGAGACGCCGCAGGGATCTGAACCTCGTCCTGGAAACCAG
mascRNA GG36-37AA (S)	GATCACTTAATACGACTCACTATAGGGGATGCTGGTGGTTGGCACTCCTGGTTTCCAGGACGAAG
mascRNA GG36-37AA (A)	TGGAGACGCCGCAGGGATCTGAACTTCGTCCTGGAAACCA
mascRNA G36A/C47T (S)	GATCACTTAATACGACTCACTATAGGGGATGCTGGTGGTTGGCACTCCTGGTTTCCAGGACGAGGTTCAGATCTCTGCGGCGTCTCCA
mascRNA G36A/C47T (A)	TGGAGACGCCGCAGAGATCTGAACCTCGTCCTGGAAACCAGGAGTGCCAACCACCAGCATCCCCTATAGTGAGTCGTATTAAGTGATC
mascRNA GG36-37AA/CC46-47TT (S)	GATCACTTAATACGACTCACTATAGGGGATGCTGGTGGTTGGCACTCCTGGTTTCCAGGACGAAGTTCAGATTTCTGCGGCGTCTCCA
mascRNA GG36-37AA/CC46-47TT (A)	TGGAGACGCCGCAGAAATCTGAACTTCGTCCTGGAAACCAGGAGTGCCAACCACCAGCATCCCCTATAGTGAGTCGTATTAAGTGATC
mascRNA Δ1 (F)	GATCACTTAATACGACTCAC
mascRNA Δ1 (R)	TGGAGACGCCGCAGG
mascRNA Δ2 (F)	GATCACTTAATACGACTCAC
mascRNA Δ2 (R)	TGGAGACGCCGCAGG
mascRNA Δ3 (F)	GATCACTTAATACGACTCAC
mascRNA Δ3 (R)	TGGAGACGCCGCAGA
mascRNA Δ4 (F)	GATCACTTAATACGACTCAC
mascRNA Δ4 (R)	GCCGCAGGGATCTGAAC

mascRNA G36A (F)	GATCACTTAATACGACTCAC
mascRNA G36A (R)	TGGAGACGCCGCAGG
mascRNA GG36-37AA (F)	GATCACTTAATACGACTCAC
mascRNA GG36-37AA (R)	TGGAGACGCCGCAGG
mascRNA G36A/C47T (F)	GATCACTTAATACGACTCAC
mascRNA G36A/C47T (R)	TGGAGACGCCGCAGAG
mascRNA GG36-37AA/CC46-47TT (F)	GATCACTTAATACGACTCAC
mascRNA GG36-37AA/CC46-47TT (R)	TGGAGACGCCGCAGAA

* F, forward; R, reverse; S, sense; A, antisense
† m, 2′-O-methyl; d, DNA
